# Desirable plant cell wall traits for higher-quality miscanthus lignocellulosic biomass

**DOI:** 10.1186/s13068-019-1426-7

**Published:** 2019-04-15

**Authors:** Ricardo M. F. da Costa, Sivakumar Pattathil, Utku Avci, Ana Winters, Michael G. Hahn, Maurice Bosch

**Affiliations:** 1 Institute of Biological, Environmental and Rural Sciences, Aberystwyth University, Plas Gogerddan, Aberystwyth, Ceredigion, SY23 3EE UK; 2 Complex Carbohydrate Research Center, University of Georgia, 315 Riverbend Rd., Athens, GA 30602-4712 USA; 3 DOE-BioEnergy Science Center (BESC), Oak Ridge National Laboratory, Oak Ridge, TN 37831 USA; 4 Present Address: Centre for Functional Ecology, Department of Life Sciences, University of Coimbra, Calçada Martim de Freitas, 3000-456 Coimbra, Portugal; 5 Present Address: Mascoma LLC (Lallemand, Inc.), 67 Etna Road, Lebanon, NH 03766 USA; 6 Present Address: Faculty of Engineering, Bioengineering Department, Recep Tayyip Erdogan University, 53100 Rize, Turkey

**Keywords:** Bioenergy, Biomass, Carbohydrate, Cell wall, Glycan, Lignin, Lignocellulose, Miscanthus, Recalcitrance

## Abstract

**Background:**

Lignocellulosic biomass from dedicated energy crops such as *Miscanthus* spp. is an important tool to combat anthropogenic climate change. However, we still do not exactly understand the sources of cell wall recalcitrance to deconstruction, which hinders the efficient biorefining of plant biomass into biofuels and bioproducts.

**Results:**

We combined detailed phenotyping, correlation studies and discriminant analyses, to identify key significantly distinct variables between miscanthus organs, genotypes and most importantly, between saccharification performances. Furthermore, for the first time in an energy crop, normalised total quantification of specific cell wall glycan epitopes is reported and correlated with saccharification.

**Conclusions:**

In stems, lignin has the greatest impact on recalcitrance. However, in leaves, matrix glycans and their decorations have determinant effects, highlighting the importance of biomass fine structures, in addition to more commonly described cell wall compositional features. The results of our interrogation of the miscanthus cell wall promote the concept that desirable cell wall traits for increased biomass quality are highly dependent on the target biorefining products. Thus, for the development of biorefining ideotypes, instead of a generalist miscanthus variety, more realistic and valuable approaches may come from defining a collection of specialised cultivars, adapted to specific conditions and purposes.

**Electronic supplementary material:**

The online version of this article (10.1186/s13068-019-1426-7) contains supplementary material, which is available to authorized users.

## Background

Following global trends, in the last decade, the World Bank has implemented a series of measures to severely limit and eventually stop financing fossil fuel-related projects. Firstly, coal-fired plants ceased to be financed [1], and more recently, the World Bank further announced it would also stop financing oil and gas exploration and extraction from 2019 onwards [2]. These decisions suggest that international monetary organisations are sending the message that fossil fuel resources are part of an antiquated technology, with reduced investment attractiveness. This shift in investment strategy also reaffirms the perception that fossil fuels pose a substantial threat to the global environment, and that weaning ourselves from these resources, as a society, will benefit the reduction of greenhouse gas emissions (GGEs) and mitigation of anthropogenic climate change. Indeed, even multinational petrochemical companies, such as Exxon and Shell, do not dispute the links between their products and anthropogenic climate change [3, 4]. In late 2018, the United Nations Intergovernmental Panel on Climate Change (IPCC) issued a special report on the benefits of limiting global warming to 1.5 °C, instead of the previous goal of 2 °C above pre-industrial levels [5]. In order to achieve this aim, the IPCC recommended that, among other measures, by 2050, up to 85% of global electricity must be supplied by renewable resources, including energy crops. In fact, the report went further as it stated that up to 7 million km^2^ of land—an area almost the size of Australia—would be needed for energy crops. Although some have argued that land-use change for the production of energy crops may increase GGEs [6], this claim is essentially valid only for first-generation corn-based ethanol production, and for the cases where forest and grassland are being diverted to biofuel production. A viable alternative to achieve a large proportion of the IPCC target of 7 million km^2^ without extensive land-use change is the utilisation of second-generation lignocellulosic feedstocks, which are non-food resources that can be produced with high yields in marginal land [7]—abandoned agricultural land, degraded land, reclaimed land and wasteland. Furthermore, a large-scale industry based on lignocellulose also represents a more sustainable alternative to petrochemical pathways, as released carbon is balanced by photosynthetic capture [8]. From an economic perspective, such a model would still be able to generate new job opportunities [9], and as novel biomass conversion processes emerge, the potential for lignocellulosic biomass valorisation is rapidly increasing [10–12].

Biomass from *Miscanthus* spp. has long been considered as a promising lignocellulosic feedstock for biorefining applications [13–16]. Reasons for this have to do with the fact that miscanthus crops have high biomass yields, wide climatic versatility and are suitable for cultivation on marginal land, while requiring very low chemical inputs [16–19]. Moreover, particularly for triploid miscanthus hybrids, these crops have vigorous growth and although there is considerable genotypic diversity, *Miscanthus* spp. generally exhibit good abiotic stress tolerance [20–22].

Plant cell walls make up most of lignocellulosic biomass, and the miscanthus cell wall, similar to that of other grass energy crops, contains large amounts of polymerised sugars, which may be used to produce biofuels and other bioproducts. However, these sugars are included in very complex molecular structures. Carbohydrates in miscanthus lignocellulosic biomass consist of cellulose, a high abundance of xylans (arabinoxylan, AX; glucuronoarabinoxylan, GAX), a low percentage of xyloglucan (XG) and mixed-linkage 1 → 3,1 → 4-β-glucan (MLG) and small amounts of pectins (homogalacturonan, HG; rhamnogalacturonan-I and -II, RG-I, RG-II) and arabinogalactan-containing polysaccharides and arabinogalactan proteins, AG, AGPs [23]. Additionally, lignin, which is a complex phenolic heteropolymer that typically comprises the second most abundant polymer in miscanthus cell walls, and acetate and hydroxycinnamates (HCAs), which occur as substituents of the main cell wall polymers, are non-carbohydrate components incorporated in miscanthus lignocellulosic biomass [23]. Understanding this plethora of cell wall components, which on their own are already complex, is further complicated by the fact that even within the same miscanthus variety, biomass from different organs or maturity stages may present substantial differences in their cell wall architecture, as our previous work has suggested [22–24]. Specifically, relative abundances of cell wall components and their fine structure are variable, and we still have limited understanding of the processes involved in interconnecting the cell wall components into different matrices according to the functional requirements of the organ and tissue they constitute [11, 25]. This immense compositional and structural complexity of the plant cell wall is deemed to have distinct impacts on biomass deconstruction, and this is the main reason why the exact basis and mechanisms of cell wall recalcitrance to deconstruction have not yet been completely understood.

Here, we present a study on the cell wall assembly of various genotypes from the two main species of miscanthus, *Miscanthus sinensis* and *M. sacchariflorus*, as well as from hybrids between the two species, including the predominant commercially grown triploid genotype of *M*. × *giganteus*. In an effort to provide fundamental knowledge about commelinoid monocot cell walls and address its resistance to deconstruction, we set out to identify cell wall traits that are simultaneously relevant for recalcitrance and distinctive between miscanthus biomasses with high or low saccharification performances. The findings of our study will help in setting and refining objectives for the breeding of miscanthus varieties with improved biorefining capabilities, thereby reducing downstream pretreatment and hydrolysis costs.

## Methods

### Plant biomass

From a previously described field trial [24], eight miscanthus accessions were selected: the predominant commercially grown variety *M. *×* giganteus* (gig01), a second *M. *×* giganteus* (hyb03; 64% *M. sinensis,* 36% *M. sacchariflorus* [26]), *M. sinensis* (sin08, sin09, sin11, sin13, sin15) and *M. sacchariflorus* (sac01). Further information on the ploidy of each accession is provided elsewhere [24]. For each genotype, a single tiller with representative thickness and minimum length of three-quarters of total plant height (excluding rhizomes and inflorescences) was randomly collected from three replicate plots. All collected tillers were harvested from mature plants: 18 weeks after emergence, when growth had mostly ceased (peak biomass); 42 weeks after emergence, when plants were senesced. After collection, samples were frozen, freeze-dried, stem and leaf (including sheath) were separated, and individually ground to a particle size between 0.18 and 0.85 mm. Subsequently, purified cell wall material (CWM) was prepared as described elsewhere [27]. Briefly, the biomass was subjected to a series of organic solvent washes, followed by α-amylase treatment. This CWM was used for all ensuing compositional analyses.

### Saccharification and monosaccharide analyses

Total monosaccharide quantitation was performed via a method based on Saeman hydrolysis, as previously described [23]. Generally, the method consisted of adding H_2_SO_4_ to the CWM samples at 30 °C for 1 h, and then diluting the hydrolysate to 4% H_2_SO_4_ (w/w) and placing it in an autoclave at 121 °C for 1 h.

Our aim was to determine the impact of cell wall features on its deconstruction; therefore, to avoid masking of recalcitrance differences, no biomass pretreatment was employed. CWM digestibility was assessed via enzyme-mediated hydrolysis and quantitation of released monosaccharides. The enzyme cocktail consisted of a mixture of Celluclast (NS 50013; cellulase) and Novozyme 188 (NS 50010; β glucosidase) at a 4:1 ratio (both obtained from Novozymes, Bagsvaerd, Denmark). Per each CWM sample the added mixture comprised of 997 μL of KOAc buffer at 0.025 M (pH = 5.6), 2.4 μL of Celluclast and 0.6 μL of Novozyme 188, with added sodium azide at 0.04% (w/v) to inhibit microbial growth. Samples were incubated at 50 °C/150 rpm for 36 h. Cellulase loadings were in excess of 14 filter paper units per gram (FPU/g) of CWM, based on a cellulase activity value of 60 FPU/mL for Celluclast. High enzyme loads ensured that enzymes were not limiting to the reaction.

The hydrolysates resulting from acid hydrolysis and enzymatic saccharification were analysed by high-performance anion-exchange chromatography with pulsed amperometric detection (HPAEC-PAD), on a CarboPac SA10 (4 × 250 mm) column, operating on a 14-min per sample, 1.5 mL min^−1^ isocratic elution, with 0.001 M KOH [23]. Calibration standards were used for monosaccharide identification and quantitation.

### Determination of lignin, acetyl and hydroxycinnamoyl esters

Lignin content determinations were performed using the acetyl bromide procedure as previously described [24]. Acetate determination was achieved by alkaline saponification (0.1 M KOH; 16 h; 21 °C/150 rpm) followed by high-performance liquid chromatography—refractive-index detection. Ester-linked hydroxycinnamic acids (HCAs) were released via alkaline saponification with 1 M KOH (16 h; 21 °C/150 rpm). Monomeric ferulic and *p*-coumaric acids were identified and quantified, using authentic standards on a reversed-phase high-performance liquid chromatography system with a diode array detector. Both procedures are described in detail elsewhere [23].

### Glycome profiling

The glycome profiling study and auxiliary phenol–sulphuric acid assays, were carried out as stated elsewhere [28, 29]. Briefly, a sequential series of increasingly harsh extractions were carried out on the CWM employing the following solutions: 0.05 M ammonium oxalate, 0.05 M sodium carbonate (with 0.5% (w/v) sodium borohydride), 1 M KOH, 4 M KOH, acidic sodium chlorite and 4 M KOH PC (post-chlorite treatment). All KOH reagents were supplemented with 1% (w/v) sodium borohydride. Each resulting cell wall extract was neutralised, dialysed and lyophilised and then screened using a suite of 155 glycan-directed monoclonal antibodies (mAbs), obtained from the Complex Carbohydrate Research Center laboratory stocks (CCRC, JIM and MAC series) or from BioSupplies (Australia) (BG1, LAMP). More details regarding the glycan-directed mAb classes are available in Additional file 1.

### Data normalisation and analysis

A previously reported reference glycome profile for the miscanthus cell wall [23] revealed that the most significant variability between the studied genotypes could be summarised using seven glycan-directed mAbs, which recognise epitopes in xyloglucans, CCRC-M87 [30]; xylans, CCRC-M154, CCRC-M144 and CCRC-M137 [31]; MLG, BG1 [32]; arabinogalactans, CCRC-M7 [33, 34] and homogalacturonan, CCRC-M38 [28]. For each sequential extract produced from each cell wall sample, of a given genotype and harvest, the net optical density (OD) of each of the seven above-mentioned mAbs was used as a measure of binding intensity of the respective mAb. These OD values were normalised to the amount of recovered carbohydrate in that particular cell wall extract. Subsequently, normalised epitope abundances from the six extracts were summed to obtain a single normalised value representing the total epitope abundance for each specific mAb (*N*). Calculations are summarised in the following equation:

$$ N_{k} = \mathop \sum \limits_{i = 1}^{6} \frac{{{\text{OD}}k_{i} \times R_{i} }}{{E_{i} }}, $$

where, for each *k* mAb (CCRC-M87, CCRC-M154, CCRC-M144, CCRC-M137, BG1, CCRC-M7, CCRC-M38), *ODk*_*i*_ is the optical density obtained for mAb *k* in extract *i* (oxalate, carbonate, 1 M KOH, 4 M KOH, chlorite and 4 M KOH PC); *R*_*i*_ is the released carbohydrate in extract *i* per g of CWM, estimated as glucose equivalents by the phenol–sulphuric acid assay (mg g^−1^ CWM); *E*_*i*_ is the carbohydrate concentration in the total CWM extract *i*, estimated as glucose equivalents, by the phenol–sulphuric acid assay (mg mL^−1^).

All descriptive statistics, ANOVA, Tukey’s tests and correlations were calculated at a 5% significance level (*α* = 0.05). Effect sizes were calculated as eta-squared statistics [35, 36]: *η*^2^ = SS_effect_/SS_total_ (where SS is the sum of squares). Agglomerative hierarchical clustering of the genotypes, according to the measured cell wall traits, was performed using the ‘hclust’ R-function (R Development Core Team, 2017).

### X-ray micro computed tomography scanning

For seven of the eight genotypes used here, a representative tiller was collected at the senesced stage. For each selected tiller, a mid-section of the third internode was taken for X-ray micro computed tomography scanning (μCT) and placed in a sample holder. The holders were loaded into the sample carousel of a μCT100 scanner (Scanco Medical, Switzerland) [37]. Internode sections were scanned with the X-ray power set at 45 kVp and 200 μA with an integration time of 200 ms. For each section, 117 slices were acquired at a resolution of 4.9 μm/pixel. Output images were processed with the Fiji software [38]: image stacks, comprising of 117 slices, were converted to an average intensity Z-projection image with auto-adjusted brightness and contrast.

### In situ immunolabeling

A single tiller cut above the rhizome was collected at the peak biomass harvest. Sampling uniformity across eight miscanthus genotypes, with varying phenotypes, was achieved by collecting leaf and stem samples from the internode located halfway through the length defined between the uppermost full-grown ligule and the base of the tiller. Smaller sections (1 mm–5 mm) were cut from the middle portion of the leaf blade and from the middle of the internode. Subsequent steps for tissue fixation, processing and epitope immunolabeling were performed as previously described [23]. Negative immunological controls (Additional file 2: D) consisted of treating sections following this same procedure, but using primary mAbs which do not bind epitopes in the tissues [39]. For certain mAbs known to only bind to de-esterified forms of the epitopes, sections were subjected to a base treatment (BT) with 0.1 M KOH (1 h followed by three washes with deionised H_2_O), before blocking and mAb application. Microscopic inspection was performed using an Eclipse 80i microscope (Nikon Inc., Melville, New York, USA) equipped with epifluorescence optics. At least three micrographs for a given mAb were captured at the same exposure time with a Nikon DS-Ri1 camera head using NIS-Elements Basic Research software (Nikon). Additional file 2 contains the full immunolabeling study using 22 mAbs.

## Results and discussion

### Miscanthus cell wall and enzymatic hydrolysis

We used an enzyme-mediated approach to determine the amenability to deconstruction of CWM from leaf and stem biomass from *M. sinensis*, *M. sacchariflorus* and two *M. *×* giganteus* genotypes. The hydrolytic mixture contained a broad spectrum of cellulolytic enzyme activities, including various cellobiohydrolases, and endo-(1 → 4)-β-glucanases; of which, some have been reported to also have xylanase activity [40, 41]. The CWM was not pretreated before enzymatic hydrolysis, to avoid minimising differences in recalcitrance between samples, and thus assess non-attenuated impacts of cell wall features on recalcitrance.

In mature miscanthus tissues (peak biomass and senesced stages), saccharification efficiencies from leaf biomass ranged between 12.4 and 23.2 GlcE (% of total glucose extracted), 2.7–8.6 XylE (% of total xylose extracted) and 2.4–8.7 AraE (% of total arabinose extracted). For stems, the values were 9.8–28.0 GlcE, 7.3–18.3 XylE and 3.5–14.0 AraE (Additional file 3). ANOVA revealed that the genotype, organ and development stage factors had significant effects on the extractability of glucose, xylose and arabinose (*P *< 0.05; Additional file 4). GlcE is typically higher in leaf biomass, whereas XylE and AraE tend to be higher in stems. However, for these three monosaccharides, saccharification efficiency generally decreases as plants mature (Additional file 3). Notwithstanding these overall trends in the extractability of glucose, xylose and arabinose, not all genotypes showed similar behaviours, suggesting genotype-specific responses to enzymatic hydrolysis.

Lignin, ferulic (FA) and *p*-coumaric (*p*CA) acids, as well as the monosaccharides glucose, xylose and arabinose, that constitute cellulose, arabinoxylans (AX) and mixed-linkage 1 → 3,1 → 4-β-glucan (MLG), are, respectively, the main components of the phenolic and carbohydrate fractions of miscanthus CWM [42, 43]. Additionally, substitution by *O*-acetyl groups, which in grass cell walls occurs primarily on AX, can affect structural integrity [44–47], namely at the level of xylan–cellulose interactions [48–50]. All these cell wall components are linked among themselves and to others, forming intertwined networks of polymers, which confer the cell wall with physiological, mechanical and chemical integrity [46, 51–53].

A previously reported reference glycan profile for miscanthus cell wall showed that certain portions of the glycome are particularly heterogeneous between different miscanthus genotypes [23]. For a more focused study of these heterogeneous regions, we chose seven glycan-directed mAbs: CCRC-M87—galactosylated xyloglucan [30]; CCRC-M154—arabinosylated xylans [31]; CCRC-M144—4-*O*-methyl glucuronic acid (Me-GlcA)-substituted xylans [31]; CCRC-M137—unmodified xylan backbone, DP ≥ 4 [31]; BG1–MLG [32]; CCRC-M7—arabinosylated (1 → 6)-β-d-galactan epitopes found in AGPs and RG-I [33, 34]; CCRC-M38—fully unesterified homogalacturonan backbone [28].

### Genotypic variation of tissue organisation and biomass quality

**Fig. 1:**
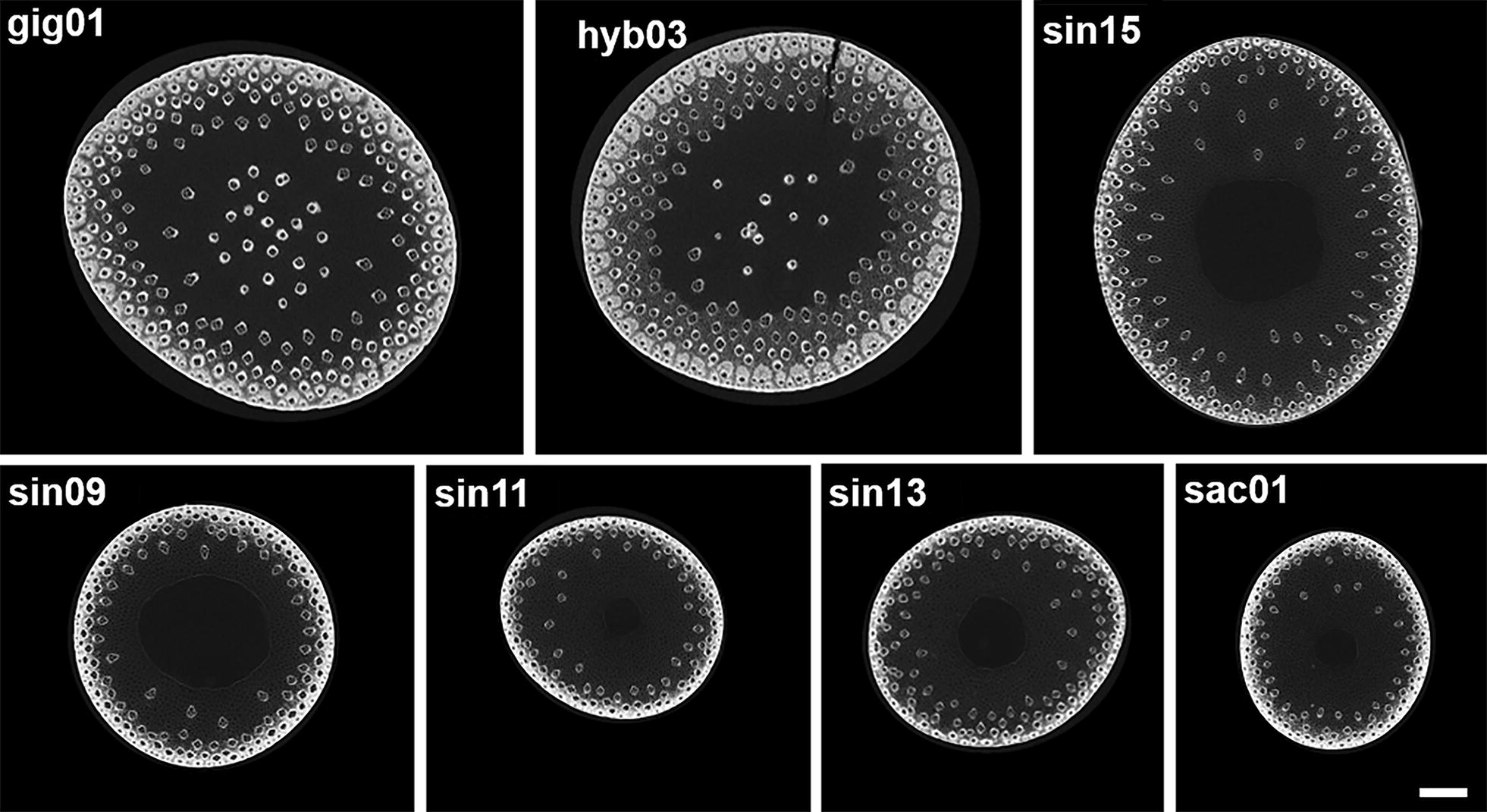
μCT scanning of senesced stem midsections from 7 of the 8 miscanthus genotypes used in this study. These data show clear differences in the stem diameter and anatomical organisation of the different genotypes, in particular for vascular bundle number and distribution, and non-hollow stems in the two *M. *×* giganteus* genotypes (gig01 and hyb03). Images are Z-projections of 117 slices. Scale bar: 1 mm

Representative images acquired by X-ray μCT of senesced miscanthus stem sections showed clear anatomical variation between different genotypes (Fig. 1). For instance, gig01 and hyb03 are characterised by the presence of vascular bundles in the pith area, whereas in the *M*. *sinensis* genotypes and in sac01, these are absent and the pith has disintegrated to form a hollow stem. Moreover, gig01 and hyb03 have similar cross-sectional areas, double that of sin09 and sin13 and three times that of sin11 and sac01. The area for sin15 cross sections was ~ 90% of those of the *M*. × *giganteus* genotypes. There is also variation in the number of vascular bundles per unit area; this measure being highest for sin15 followed by gig01 and hyb03 with the lowest value for sac01.

**Fig. 2:**
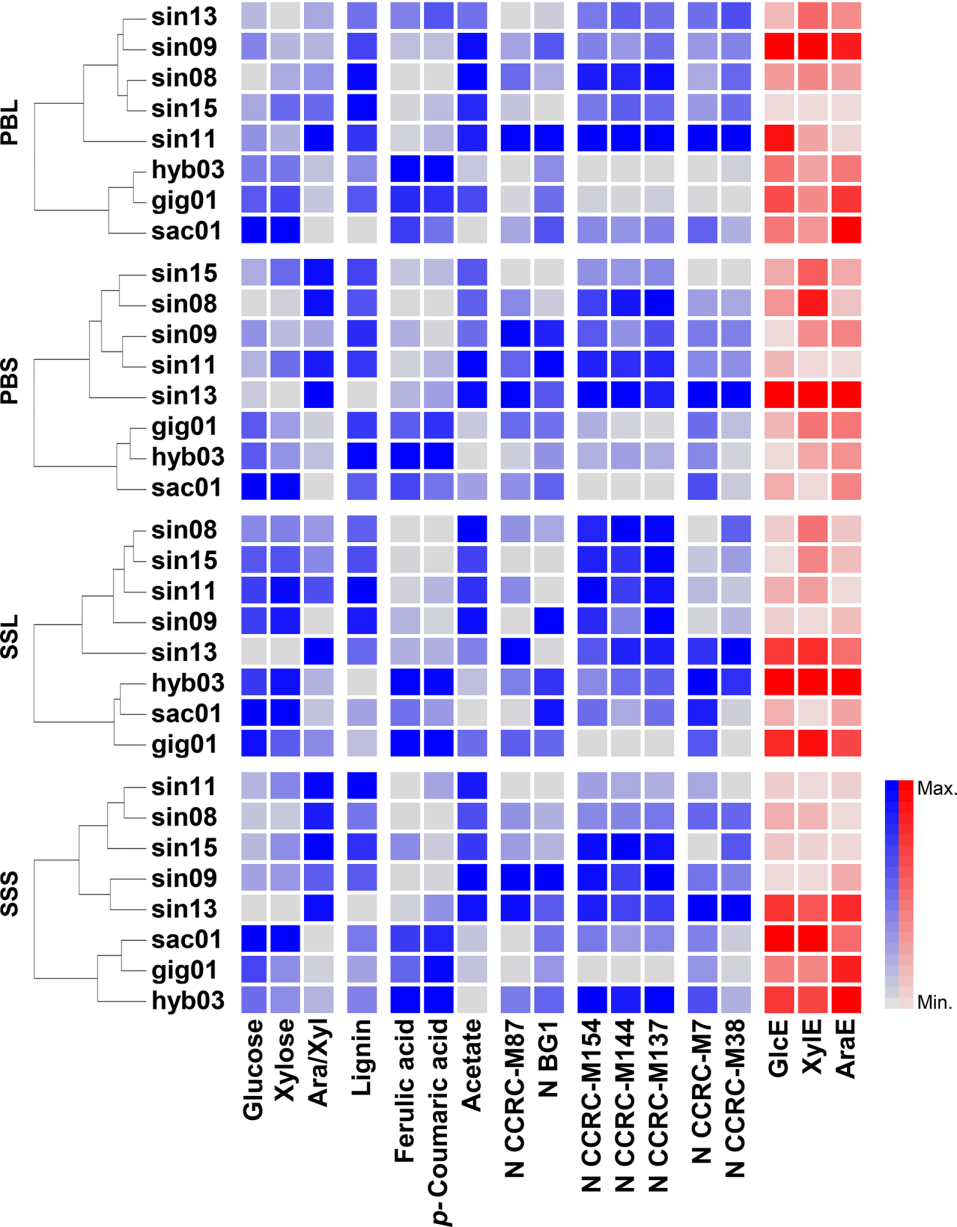
Clustering of 8 miscanthus genotypes, based on the cell wall traits from their leaves (L) and stems (S), harvested at peak biomass (PB) and senesced (SS) stages. For a given dendrogram, the colour intensities within each row of the adjacent heatmap, represent relative abundances of corresponding cell wall traits in that genotype. Shades of red represent saccharification efficiency indices: the percentages of total glucose (GlcE), xylose (XylE) and arabinose (AraE) released upon enzymatic saccharification. Cell wall compositional features are represented in shades of blue. Ara/Xyl, is the ratio of arabinose to xylose determined in the cell wall material (CWM). *N* designates the normalised value representing the total epitope abundance (OD/g of CWM) for specific mAbs: xyloglucans (CCRC-M87, galactosylated xyloglucan), glucan (BG1, MLG), xylans (CCRC-M137; CCRC-M144; CCRC-M154) and pectins (CCRC-M7, RG-I/AG; CCRC-M38, homogalacturonan backbone). Only cell wall composition data (blue), and not the three saccharification-related variables (red), were considered for genotype classification, which was performed via agglomerative hierarchical clustering, using the ‘hclust’ R-function. More detail regarding the glycan-directed mAb classes is available in Additional file 1. Genotype sin13 often showed compositional traits misaligned with the remaining *M. sinensis* genotypes, as its biomass was typically made up of softer tissues than those of other genotypes. This is likely to be caused by phenotypical properties of sin13 at the level of an intrinsic and distinct developmental gradient that occurs within the plant anatomy

The data derived from determining the relative abundances of the seven most discriminant glycan epitopes detected by the above-mentioned mAbs, and of glucose, xylose, arabinose, lignin, FA, *p*CA and acetate, have allowed to pinpoint desirable and undesirable traits in the miscanthus lignocellulosic crop for optimal saccharification. Hierarchical clustering, employing all these cell wall traits, provided better understanding of how the miscanthus genotypes differ from each other (Fig. 2). The resulting dendrogram shows that the main clustering between the different miscanthus genotypes caused the separation of *M. sinensis* (sin08, sin09, sin11, sin13, sin15) from *M*. × *giganteus* (gig01, hyb03) and *M*. *sacchariflorus* (sac01). The formation of these two main groups clearly suggests that biomass from *M. sinensis* is compositionally distinct from that of other genotypes included in this study. As a trend, *M*. × *giganteus* and *M. sacchariflorus* genotypes have higher relative abundances of ferulic and *p*-coumaric acids, glucose and xylose, except for their senesced leaf biomass, where relative content in these monosaccharides is more uniform between the two clusters. Most of the arabinose in grass cell walls is found bound to xylan backbones, forming arabinoxylan (AX), which is the most abundant arabinosylated polysaccharide in miscanthus cell wall [23, 54]. Arabinose/xylose ratios are frequently used as indicators of the degree of xylan arabinosylation [55], and in *M*. *sinensis* biomass, these ratios tend to be higher than in *M. *×* giganteus* and *M. sacchariflorus* genotypes. Similarly, the relative abundance of lignin, acetate and of xylan epitopes (probed by CCRC-M137, CCRC-M144 and CCRC-M154) are typically higher in *M*. *sinensis*.

Only cell wall compositional and structural traits were considered for the clustering analysis. Yet, by analysing the associated saccharification efficiency indices (GlcE, XylE and AraE), represented in shades of red (Fig. 2), we see that senesced non-sinensis genotypes, hyb03 and gig01, tend to display relatively higher saccharification efficiencies. To obtain a deeper understanding of how varying compositional and structural cell wall features impact on saccharification, four genotypes were chosen to include two representatives from each group arising from the clustering analysis: hyb03, sac01, sin08 and sin11.

**Fig. 3:**
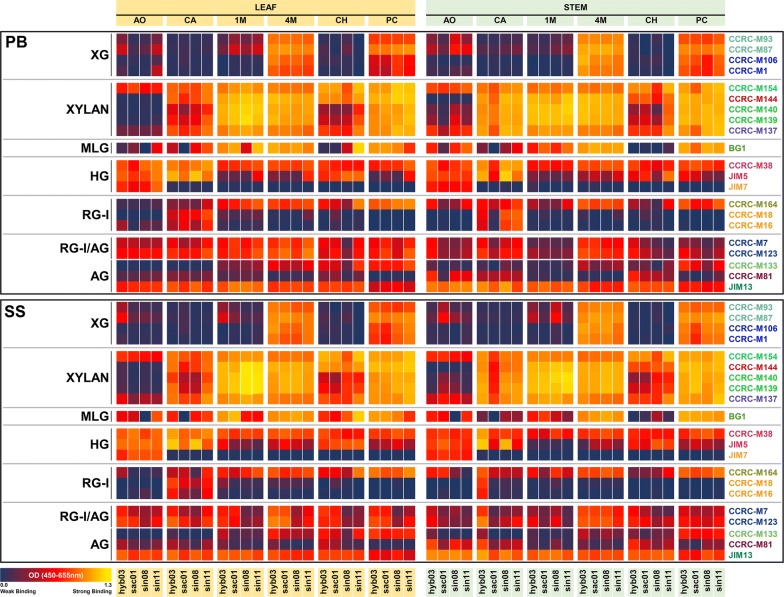
Detail of the glycome profile of leaf and stem cell wall biomass from 4 miscanthus genotypes, using 21 of the 155 glycan-directed mAbs. Cell wall fractionation was achieved by sequentially employing ammonium oxalate, AO; sodium carbonate, CA; 1 M KOH, 1 M; 4 M KOH, 4 M; sodium chlorite, CH and 4 M KOH post-chlorite treatment, PC. Clear differences are visible in cell wall glycans between the various genotypes, at identical developmental stages and organs. The different colours of the mAb names to the right refer to their glycan-binding subclasses, which are further elucidated elsewhere [28, 30–32] and in Additional file 1. Antibody binding strength based on optical density (OD) is presented as a colour gradient ranging from dark blue (no binding: OD = 0.0) and red (intermediate binding) to yellow (strongest binding: OD = 1.3). For complete glycome profiles for all tested genotypes and 155 mAbs, including the amounts of extracted carbohydrate in each sequential extract, refer to Additional file 5. PB, peak biomass; and SS, senesced stages; XG, xyloglucan; MLG, mixed-linkage glucan; HG, homogalacturonan; RG-I, rhamnogalacturonan-I; AG, arabinogalactan; RG-I/AG, AG side chains of RG-I

**Fig. 4:**
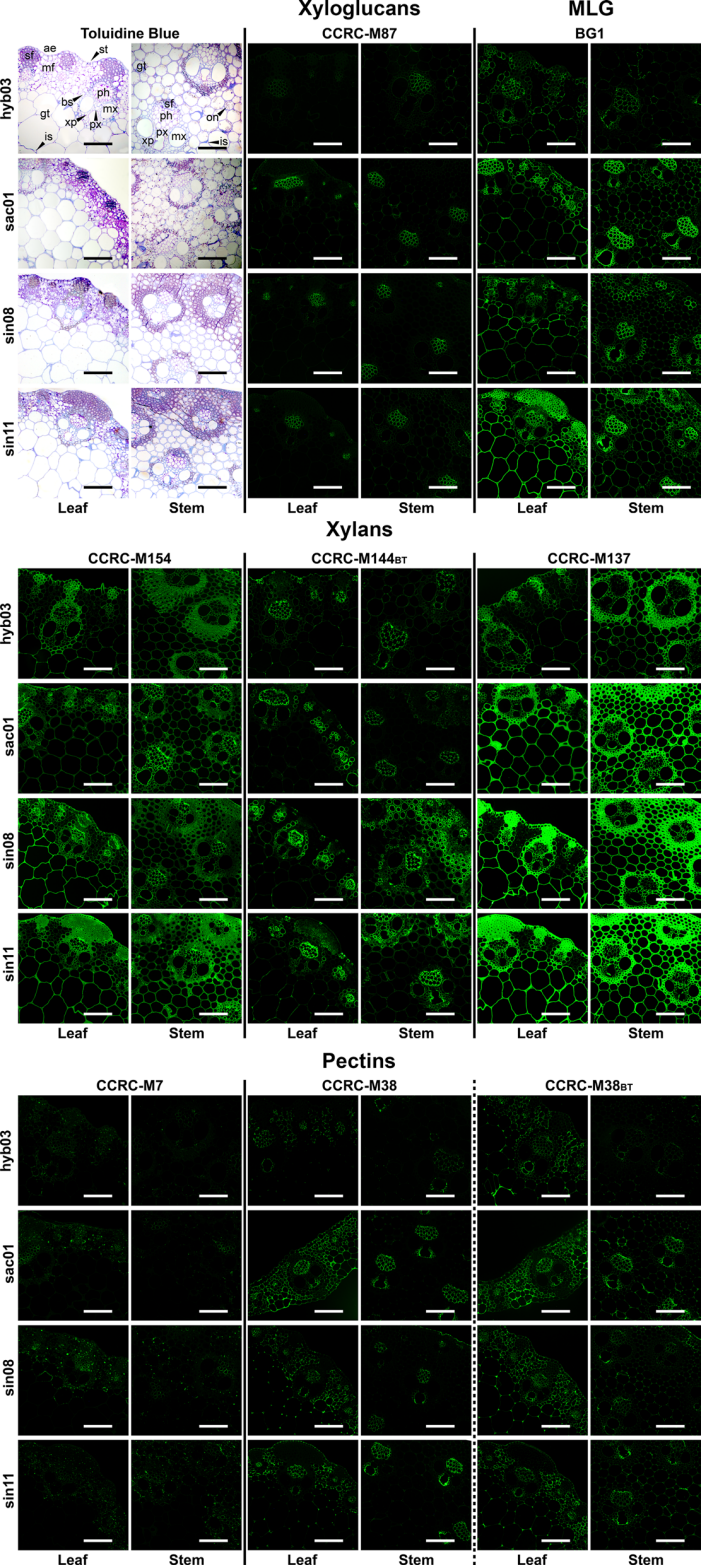
Immunofluorescent labelling of cell wall glycan epitopes in transverse sections from leaves and stems from 4 miscanthus genotypes. Immunolabeling studies were preceded by microscopic inspection of sections stained with toluidine blue to characterise their histological complexity. Monoclonal antibodies (mAbs) used in the study are directed at xyloglucan, MLG, xylan and pectin epitopes. More information on these and other mAbs is provided in Table 1 and in Additional file 1. A base treatment (BT) with 0.1 M KOH, which removed ester-linked substituents from the cell wall, was used together with CCRC-M144 and CCRC-M38. The non-base-treated control of CCRC-M155, as well as the complete immunolabeling study (total of 22 glycan-directed mAbs and 8 genotypes), is available in Additional file 2. ae, abaxial surface epidermis; bs, bundle sheath; on, organelle; gt, parenchymatous ground tissue; is, intercellular space; mf, mesophyll cells; mx, metaxylem; ph, phloem; px, protoxylem; sf, sclerenchyma fibres; st, stomatal complex; xp, xylem parenchyma. Scale bars: 100 µm

Previously determined information about the most variable portions of miscanthus cell wall glycome [23] was used to reduce the number of employed mAbs to 21. By only including these most heterogeneous portions of the glycome, the analysis of individual genotypes became more objective and targeted. Glycome profiling heatmaps (Fig. 3) and in situ immunolabeling of cell wall glycan epitopes (Fig. 4; Table 1) show further detail of the binding intensity of these glycan-directed mAbs. For all genotypes, the higher amount of carbohydrate was extracted with 1 M KOH during the sequential extraction, followed by 4 M KOH (Additional file 5). Epitopes in xyloglucan polymers, probed with CCRC-M87 showed strong labelling of the phloem, in most genotypes, except for hyb03. MLG immunolabeling with BG1 was visible throughout the tissue sections, particularly in vascular bundles and in bundle sheaths, including the cell wall of lignified sclerenchyma tissues of *M. sinensis* genotypes. Coincidentally, during glycome profiling, the binding intensity of BG1 was markedly higher in the chlorite extract from leaves of sin08 and sin11, which suggests higher abundances of lignin-associated MLG in these genotypes (Fig. 3). Probing of xylan epitopes showed distinct labelling patterns, according to the mAb and to the genotype under study, indicating xylan heterogeneity. Ester-linked substituents can be an important aspect of these differences, as suggested by employing CCRC-M144 together with a 0.1 M KOH treatment to cleave ester bonds (Fig. 4; Additional file 2: D, E). Ester-linked acetyl and hydroxycinnamoyl substituents are known to cause significant differences in biomass saccharification, and indeed, we saw that *M. sinensis* genotypes have relatively lower proportions of HCAs (Fig. 2). Further evidence of the distinct fine structure properties between the genotypes comes from probing homogalacturonan epitopes with CCRC-M38, with and without the 0.1 M KOH base treatment (Fig. 4), as the increase in labelling after alkaline saponification was more obvious in *M. sinensis* genotypes, particularly for stem sections.

**Table 1:** Cell wall glycan-directed monoclonal antibodies (mAbs) used in the study of in situ immunolabeling of miscanthus leaf and stem tissues

mAb	Epitope
CCRC-M87	Galactosylated xyloglucan [30]
BG1	Mixed-linkage (1 → 3, 1 → 4)-β-glucan [32]
CCRC-M154	Arabinosylated xylan [31]
CCRC-M144^BT^	Me-GlcA substituted xylan [28, 31]
CCRC-M137	Unmodified xylan backbone (DP ≥ 4) [31]
CCRC-M7	Arabinosylated (1 → 6)-β-d-galactan found in AGPs and RG-I [33, 34]
CCRC-M38^BT^	Unesterified homogalacturonan backbone [28]

Further information on the mAbs used here may be found in Additional file 1

^BT^ Also used in combination with a 0.1 M KOH base treatment of the sections before immunolabeling

The relevance of slight differences in glycan epitope structure and distribution may be associated with their involvement in providing structural reinforcement, or protection against external attack. Examples of this are epitopes occurring in xyloglucans (CCRC-M87) and in xylans (CCRC-M137 and CCRC-M144), which occur in corners of intercellular spaces and on the walls of the epidermis and cuticle (Fig. 4). These epitopes are only substantially removed from the cell wall with the harshest extractants, 1 M or 4 M KOH (Fig. 3). It is possible that these epitopes are integrated in glycans that enhance tissue resistance to deconstruction. Thus, higher abundances of such epitopes may result in higher recalcitrance of cell wall biomass fractions containing such polysaccharide structures.

### Associations between cell wall traits and their influence on recalcitrance

**Fig. 5:**
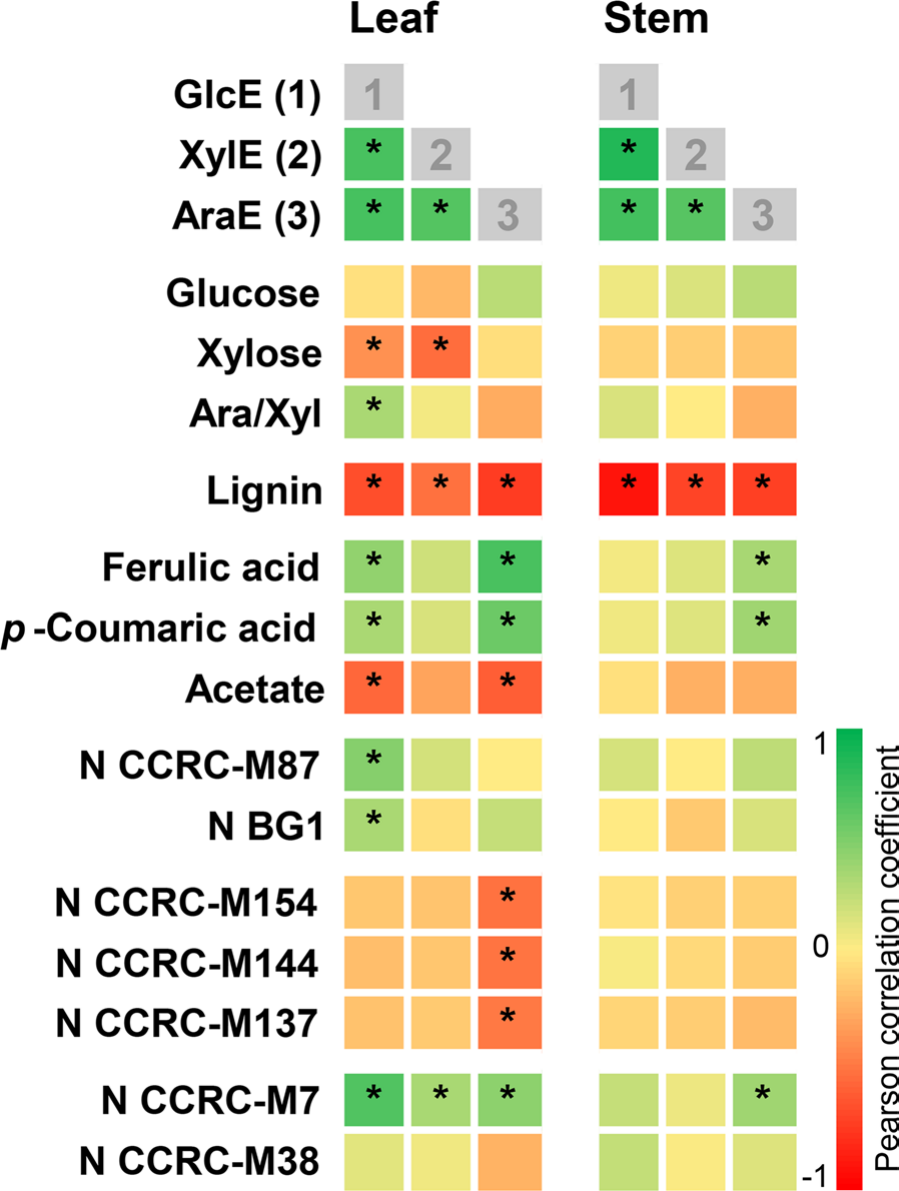
Correlation matrix between measured cell wall compositional features and enzymatic saccharification efficiency of glucose (GlcE), xylose (XylE), arabinose (AraE), in miscanthus biomass harvested at the peak biomass and senesced stages. Values for GlcE, XylE and AraE consist of the percentages of the total content of the corresponding monosaccharide that exists in the cell wall (% total glucose; % total xylose; % total arabinose), released upon enzymatic saccharification. Pearson correlation coefficients are presented as a colour gradient ranging from red to green. Statistically significant correlations (*P *≤ 0.05, *n* = 32) are identified with a ‘*’. For the eight miscanthus genotypes, GlcE, XylE, AraE, Glucose, xylose, lignin, ester-linked ferulic and *p*-coumaric acids and acetate were measured as % cell wall material (CWM). Ara/Xyl is the ratio of arabinose to xylose determined in the CWM. N designates the normalised value representing the total epitope abundance (OD/g of CWM) for specific mAbs: xyloglucans (CCRC-M87, galactosylated xyloglucan), glucan (BG1, MLG), xylans (CCRC-M137; CCRC-M144; CCRC-M154) and pectins (CCRC-M7, RG-I/AG; CCRC-M38, homogalacturonan backbone). More detail regarding the glycan-directed mAb classes is available in Additional file 1. The full correlation matrix and associated *P* values are shown in Additional file 6

A correlation matrix was constructed with the aim of assessing how the three saccharification efficiency indices (GlcE, XylE and AraE) are associated with cell wall traits. In stem and in leaf biomass, these indices are significantly and positively correlated to each other (Fig. 5). Nevertheless, in different types of biomass, distinct sets of factors can affect saccharification efficiency. Specifically, saccharification of leaves is influenced by a distinct and more complex set of cell wall traits compared to stems.

None of the saccharification indices is significantly correlated with total glucose content in the cell wall. However, GlcE from leaves is significantly and positively correlated with the relative abundances of epitopes recognised by CCRC-M87 (*r *= 0.50*, P *< 0.05) and BG1 (*r *= 0.36*, P *< 0.05), which, respectively, occur in xyloglucan and in MLG polymers. By contrast, negative correlations occur between GlcE and XylE and total xylose content in both organs, while in leaves, AraE is negatively associated with the abundance of xylan-directed mAbs (CCRC-M137, CCRC-M144 and CCRC-M154) (Fig. 5; Additional file 6: A). Glucose in grass cell walls primarily derives from cellulose, therefore our data suggest that higher amounts of cellulose may be detrimental for saccharification efficiency. Indeed, reports have suggested that polysaccharides tightly associated with cellulose are shielded from enzymatic attack by AX and MLG [56], and higher amounts of cellulose may present more structured obstacles to enzyme action [57]. CCRC-M87 and BG1 epitopes are more abundant in leaf than in stem biomass (Additional file 3), and as the employed enzymatic cocktail has primarily cellulase and β-glucosidase activity, higher proportions of non-cellulosic glucans in leaves may allow for more efficient hydrolysis of the cell wall. Conversely, as the enzymatic cocktail only has minimal xylanase activity, the coating of cellulose microfibrils with xylans may be one reason for negative correlations between saccharification, and xylose and xylan epitope abundances. Moreover, it is known that lower hemicellulose contents are associated with more effective cellulose deconstruction [58], and that several xylooligomers may inhibit enzymatic hydrolysis of glucan and xylan [59, 60]. Arabinose/xylose ratios are significantly and positively correlated with GlcE in leaves (*r *= 0.36, *P *< 0.05). In stems, these ratios are typically lower (Additional file 3). Highly substituted AXs are more abundant in the primary cell wall, while more linear xylans are often associated with lignification and secondary cell walls [61]. Experimentally, high alkali concentrations are needed for the extraction of unsubstituted xylans [53], and higher xylan arabinosylation enhances enzymatic digestibility [62]. Furthermore, the abundance of arabinogalactan epitopes probed by CCRC-M7 is significantly and positively correlated with saccharification efficiencies in both studied miscanthus organs, albeit more predominantly in leaves (Fig. 5). Similarly, in sodium carbonate and 4 M KOH extracts from leaf biomass, the abundances of CCRC-M144 and CCRC-M154 epitopes are significantly and positively correlated with GlcE and XylE (Additional file 6: C). By contrast, in 1 M KOH extracts from leaves, the correlation between the abundances of CCRC-M144 and CCRC-M154 epitopes is negative (Additional file 6: C). Altogether, these observations strongly suggest that saccharification may benefit from higher degrees of xylan glycosyl modification. However, this association is likely to be more complex than a direct association between xylan ornamentation and saccharification efficiency. Indeed, the compositional and structural nature of the parent glycoforms must be considered for optimal lignocellulose biorefining. Particularly, as we observed that acetyl and hydroxycinnamoyl ornamentation are distinctly correlated to saccharification efficiency. Acetyl and the hydroxycinnamoyl (*p*CA and FA) are typically the most abundant ester-linked xylan substituents in miscanthus cell wall [63–66]. Significant and negative correlations were seen between determined acetate content and saccharification efficiency of glucose (*r *= − 0.54, *P *< 0.05) and arabinose (*r *= − 0.58, *P *< 0.05) in leaf biomass (Fig. 5; Additional file 6: A). It is likely that acetylation of glycosyl residues of polysaccharides creates steric hindrance for binding of many hydrolytic enzymes, which limits the extent of hydrolysis [45, 47, 67]. Furthermore, it has been reported that the pattern of xylan acetylation may influence how xylan interacts with cellulose in secondary cell walls [50, 68], and that chemical deacetylation of cell wall biomass substantially improves saccharification [69–72].

In grass cell walls, FA is typically found ester-linked to arabinosyl substituents of AX [44, 73, 74], where it may simultaneously ether-link to lignin monomers [65, 75], or even form dimers and other oligomers, which crosslink carbohydrate chains [48, 52]. Here, we have determined the amounts of ester-linked monomeric FA, which are significantly and positively correlated with the saccharification efficiency of glucose (*r *= 0.45, *P *< 0.05) and arabinose (*r *= 0.73, *P *< 0.05) in leaf biomass and of arabinose (*r *= 0.37, *P *< 0.05) in stems (Fig. 5; Additional file 6: A, B). Ester-linked FA monomers may be oxidatively coupled, forming dimers and oligomers which crosslink AX polymers and produce tighter molecular structures in grasses [44, 65, 76, 77]. Thus, it may be hypothesised that in cell walls where higher abundances of monomeric ester-linked FA occur, lower proportions of these monomers have yet been coupled, leading to less recalcitrant structures, than those where FA-mediated cross-linking is more abundant. Hence providing an explanation for the positive correlations observed between ester-linked FA monomers and the saccharification indicators.

The abundance of MLG epitopes probed by BG1 in sodium chlorite extracts from leaves is negatively correlated with GlcE (*r *= − 0.43, *P *< 0.05) and XylE (*r *= − 0.50, *P *< 0.05) (Additional file 6: C). However, in 4 M KOH extracts from leaves, BG1 epitope abundance appears to be beneficial to GlcE (*r *= 0.65, *P *< 0.05) and XylE (*r *= 0.71, *P *< 0.05). As sodium chlorite removes lignin from the cell wall, glycan epitopes identified in sodium chlorite extracts, including those bound by BG1, may belong to MLG populations somehow associated with lignin. This suggests a structural role for MLG, and indeed it has been reported that the presence of MLG in mature tissues provides cell wall strengthening [78–80]. In fact, in rice it has been reported that MLG depletion is accompanied by a lignin content increase, likely as compensation for the lack of MLG [79].

Pectin epitope abundances probed by CCRC-M7 and CCRC-M38 did not show any significant negative correlation with saccharification indices in the extracts obtained with the less stringent extractants. Instead, for both mAbs, epitope abundances in 4 M KOH extracts from leaf cell wall are significantly and positively correlated with GlcE (*r *> 0.74, *P *< 0.05) and XylE (*r *> 0.58, *P *< 0.05). However, in switchgrass, rice and poplar, biomass yields and sugar release were improved in plants engineered to reduce expression of a pectin biosynthesis gene, presumably due to reduced homogalacturonan and RG-II cross-linking in the cell wall [81]. Therefore, the contribution of pectins to cell wall assembly and biomass conversion should not be considered a secondary factor when developing miscanthus varieties for optimised bioconversion.

The abundance of epitopes released after delignification (4 M KOH PC) generally have negative Pearson coefficients, demonstrating negative associations with saccharification performance (despite not statistically significant; Additional file 6: C). In 1 M KOH extracts, xylan epitopes CCRC-137, CCRC-M144 and CCRC-M154 are negatively correlated with GlcE (*r *< − 0.52, *P *< 0.05) and XylE (*r *< − 0.45, *P *< 0.05) in leaves. However, in 4 M KOH extracts, epitope abundances for CCRC-137, CCRC-M144, CCRC-M154, CCRC-M87 and BG1 are positively correlated with GlcE (*r *> 0.65, *P *< 0.05) and XylE (*r *> 0.70, *P *< 0.05) (Additional file 6: C). This supports the idea that a certain epitope probed by the same mAb in different extracts can occur in distinct glycoforms that exist in the cell wall for each polymer type, and are capable of conferring varying degrees of recalcitrance. Indeed, reports have suggested that some glycans may occur in the cell wall simultaneously as polysaccharides and proteoglycans, and that a given kind of matrix polysaccharide could encompass various distinctive polymers, each with unique roles in the primary or secondary cell walls of different cell types [81–83]. Altogether, these observations highlight the high relevance of the cell wall glycome for leaf biomass saccharification.

In stems, lignin is the only cell wall feature negatively affecting GlcE and XylE (Fig. 5). This negative effect on saccharification is mainly due to lignin–carbohydrate complexes which form barriers and cause non-productive binding with hydrolytic enzymes, limiting accessibility to cell wall core polysaccharides and inhibiting hydrolysis [84]. However, even though the negative effects of lignin on recalcitrance are well known, the structural complexity of lignin confuses our understanding of underlying relations between lignin structure and recalcitrance [11, 85]. Indeed, lignin structure, instead of lignin content, is what seems to affect recalcitrance the most, as demonstrated by improved digestibility in biomass containing lignins with more labile linkages [86].

### Saccharification performances and biomass quality

**Fig. 6:**
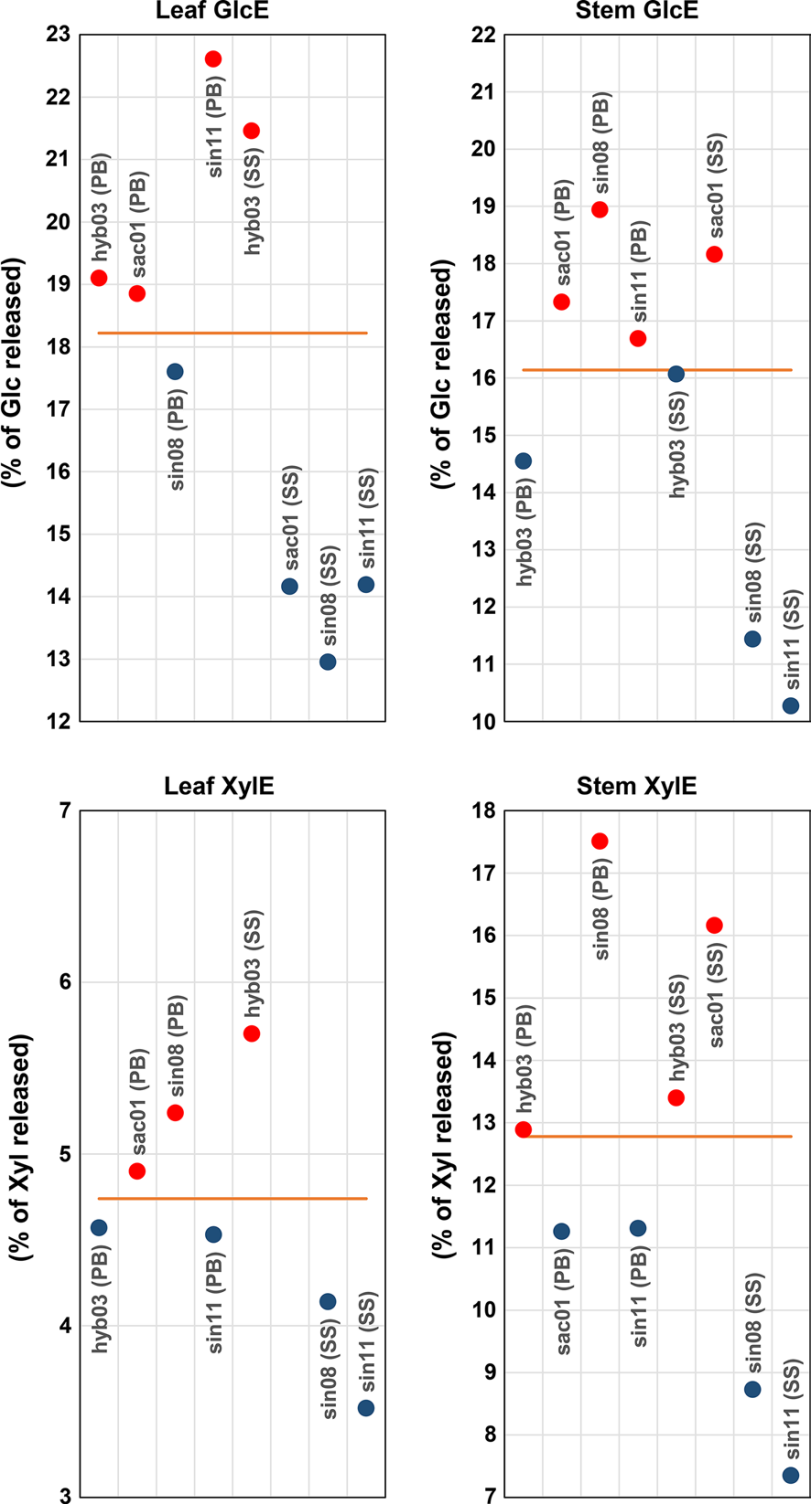
High (red) and low (blue) yielders of glucose (GlcE) and xylose (XylE), upon enzymatic saccharification. Values in the *y*-axes are the percentages of the total content of the corresponding monosaccharide that exists in the cell wall (% total glucose; % total xylose). Only the subgroup of 4 miscanthus genotypes is shown. However, the orange horizontal line represents the median of the values obtained for the 8 original miscanthus genotypes. For further details, see Additional file 7. PB, peak biomass; SS, senesced stage

To elucidate which cell wall features are desirable for optimal saccharification, we followed a categorical approach to discern between biomasses displaying “good” or “bad” conversion performances. However, the rankings seen for GlcE did not coincide with those of XylE. Furthermore, a given genotype that ranked high or low in leaf biomass did not necessarily have a comparable saccharification performance in stem. This lack of agreement between leaf and stem indicates that distinct mechanisms dictate recalcitrance to sugar release, depending on the sugar (glucose or xylose) and plant organ (leaf or stem). Leaf and stem CWM samples were therefore binarily classified as high or low yielders based on the percentage of glucose or xylose released upon enzymatic saccharification, with the median of all saccharification efficiency values representing the threshold dividing the two classes (Fig. 6).

**Fig. 7:**
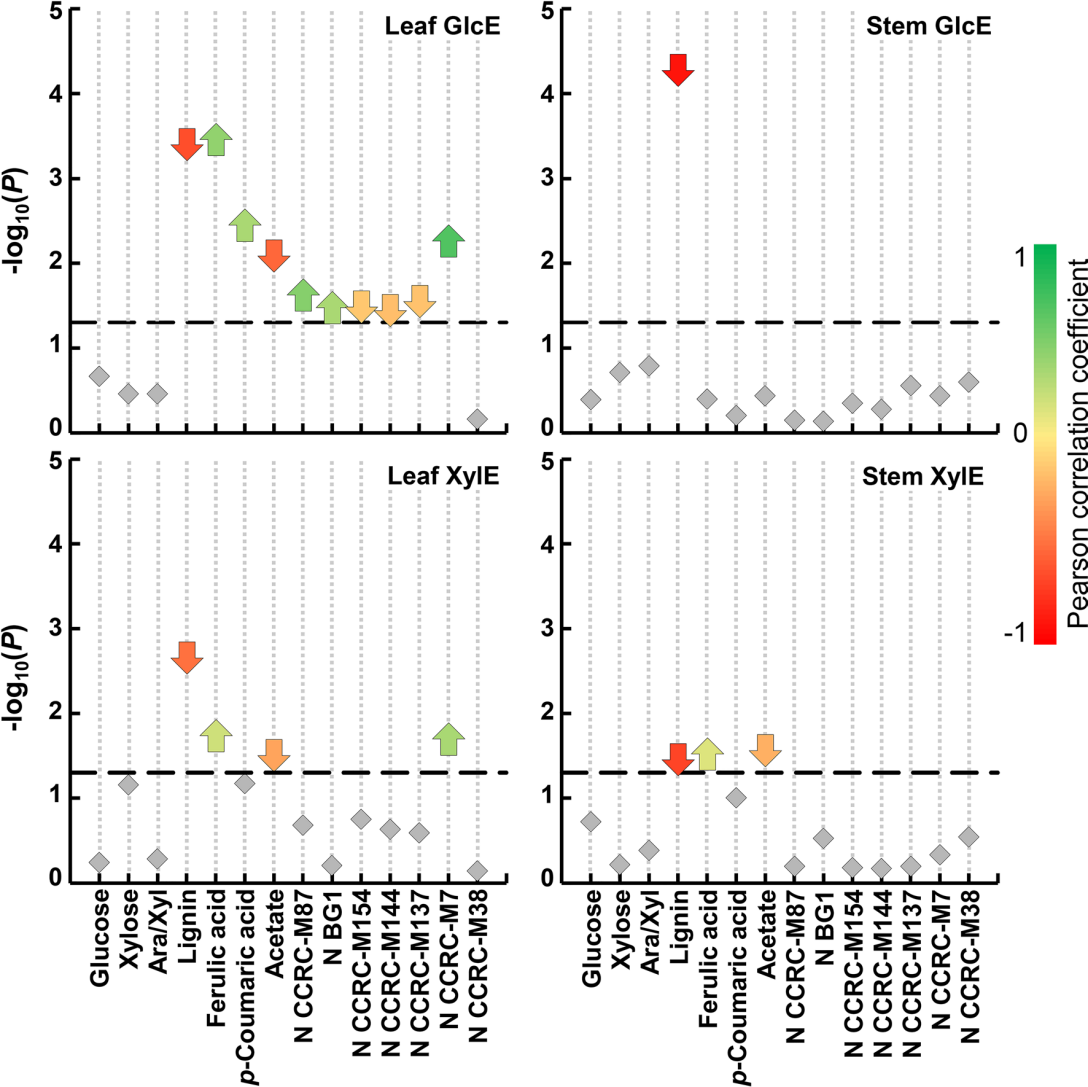
Discriminant features between high and low yielders of glucose (GlcE) or xylose (XylE), upon enzymatic saccharification of mature miscanthus biomass, detected by *t* tests (*α* = 0.05). High yielders, refer to the top 50% glucose or xylose saccharification efficiency values, whereas low yielders are the bottom 50%. Arrows represent significantly different features, i.e. when *P *≤ 0.05 (− log_10_ 0.05 ≈ 1.30). Upward pointing arrows represent features positively correlated with the saccharification efficiency, whereas downward pointing ones are negatively correlated with saccharification efficiency. The colour of the arrows indicates the Pearson correlation coefficient (as in Fig. 5). Box and whisker plots of the distribution of biomass compositional measurements are shown in Additional file 7. Ara/Xyl, arabinose-to-xylose ratio; N is a normalised value representing the total epitope abundance for each monoclonal antibody (mAb; more details in Additional file 1)

Paired-sample t-tests (Additional file 7), in combination with previously determined correlation coefficients (Fig. 5), allowed the determination of cell wall traits significantly different between “good” and “bad” performers, while simultaneously assessing the impact of these differences on saccharification efficiency (Fig. 7). High saccharification efficiencies from leaf biomass seem to depend on more features than those affecting stem biomass. This is in agreement with our previous reports, suggesting that leaf and stem cell wall assembly is regulated by distinct mechanisms, which are translated into very distinct saccharification performances [22–24]. Lignin and acetate are features negatively affecting saccharification and are significantly different between the high and low performing classes. Particularly for GlcE, the abundance of xylan epitopes is also a negative discriminant feature affecting enzymatic hydrolysis. In stem biomass, acetate and lignin contents are negative discriminant features affecting XylE, but lignin is the only cell wall trait that is significantly different between the high and low glucose yielders (Fig. 7). Despite this, we cannot presume that lignin is the only recalcitrance-enhancing agent in stem biomass. Indeed, in switchgrass, although lignin content is generally negatively correlated with saccharification, it is not the sole contributor to recalcitrance across diverse anatomical features [87].

### Desirable cell wall traits for ideotype breeding

To improve lignocellulosic crop performance, the whole plant is the final object of research. Plant growth depends on potentially resource-competing, morphological, physiological and biochemical processes, which are often hard to consider as a whole when trying to decipher a desirable phenotype for improved performance. The proposal of such phenotypes consists of the ideotype approach [88, 89], which in the context of lignocellulosic crops should consist of a cultivar that performs in a predictable manner, leading to optimal biorefining conversion and life-cycle assessment performances, while maintaining plant integrity. The discriminant cell wall traits between high and low saccharification efficiency performers (Fig. 7) helped us define which biomass traits are desirable in a miscanthus ideotype for biorefining.

Lignin is a recalcitrance-enhancing factor, in all types of miscanthus biomass tested. However, the negative correlation between lignin and saccharification efficiency is likely a consequence of lignin molecular structure instead of its overall content. Lignification is a flexible process [90], with the potential of constructing lignocellulosic crops with less recalcitrant lignins. Approaches to achieve this can vary from selecting plants enriched in syringyl units [10], which form more easily cleaved β-*O*-4 inter-unit linkages, to the generation of plants that incorporate HCA-related (FA and *p*CA) conjugates into lignin [86, 91], yielding lignin with elevated ester linkages amenable to mild alkaline depolymerisation. Numerous economic benefits exist for maintaining normal *in planta* lignin levels, while reducing its recalcitrance, particularly as novel conversion pathways emerge for lignin utilisation [11]. Furthermore, refinement of biomass downstream processing has enhanced lignin recovery, which, coupled with genetic engineering, may enable new uses for lignin [10, 92]. Additionally, a recent breakthrough process for lignin depolymerisation may improve the yields of low molecular weight aromatic monomers with high potential industrial value [12]. These technical advances may eventually lead to cost-effective utilisation of lignin-derived chemicals for parallel biomaterial production, which in turn will add value to lignocellulosic biomass applications, and thus enhance the economic viability of lignocellulosic biofuels.

Previous studies have reported cell wall composition and bioconversion traits in miscanthus aimed at improving biomass quality and facilitating its use as feedstock for biofuel production [93]. However, cell wall acetylation is often not considered as a trait with a substantial effect on cell wall recalcitrance. Here, we demonstrate that acetylation has a negative influence on cell wall saccharification and is a discriminant feature between high and low yielders (Fig. 7). In grass primary walls, most acetylation occurs in AX [47], where it partly contributes to the interaction with cellulose and lignin [50, 68, 94, 95]. Deficiencies in *Arabidopsis* mutants with reduced acetylation suggested a fundamental role of acetylation in cell wall mechanical integrity [96, 97]. Ultimately, acetate can simultaneously inhibit saccharification and fermentation [98, 99], by causing steric hindrance of hydrolytic enzymes [45, 47, 67], and by becoming toxic to fermenting microbes [46, 100]. In agreement, aspen plants with reduced xylan acetylation showed 25% higher glucose saccharification yield compared with wild types [101]. Our current knowledge still does not allow inferences about the repercussions of merely reducing *in planta* cell wall acetylation on the viability and saccharification performance of a poalean lignocellulosic crop. Nonetheless, our data clearly show that acetylation is significantly correlated with the saccharification of miscanthus biomass, and its impact should be further addressed within crop improvement programmes.

Glucose content is higher in CWM from miscanthus stems than in leaves, but glucose determinations, and therefore cellulose content is not significantly correlated with saccharification efficiency (Fig. 5). The reason for these observations is not clear, but they do indicate complex relationships between glucose content and sugar extractability. Our findings corroborate the concept that alongside relative abundances, the structure of the cell wall and how polymers interconnect, must be considered for the improvement of lignocellulosic feedstocks such as miscanthus. Higher abundance of cellulose may in fact enhance recalcitrance, because of its crystalline nature [11, 102], and it has been reported that reduction of cellulose crystallinity results in more efficient cellulase action [103]. Therefore, breeding for miscanthus varieties with increased cellulose content may not necessarily lead to better saccharification performance.

The design of a miscanthus ideotype for biorefining should also consider the abundance of certain glycan epitopes, which based on their in situ location (Fig. 4), may be integrated in polysaccharide structures involved in providing structural reinforcement (e.g. corners of intercellular spaces), or protection against external attack (e.g. epidermal cells and cuticles). Higher abundances of such epitopes may be an indicator of higher recalcitrance of the polymers where they are included. Therefore, the study of glycan distribution may provide further insights into which glycan epitopes have more relevant effects on saccharification.

Leaves are mostly composed of mesophyll and large parenchymatous cells in the ground tissue, with typically thinner cell walls than in stems. It is likely that these structural features are more amenable to hydrolytic enzyme access, and thus sugar release. We have previously suggested that leaf-to-stem ratios may influence miscanthus biorefining performances [24]. Here, we also show that mean GlcE is higher in leaves than in stems (Additional file 3). Therefore, we argue that merely increasing overall biomass yields should not be the main strategy for lignocellulosic crop improvement, as high biomass producers may not always be the most cost-effective varieties, and high carbohydrate content is not always synonymous with high saccharification efficiency. Thus, attempts to generate less recalcitrant plant varieties, namely by increasing leaf biomass proportions, even if at the cost of total biomass yield reductions, could be a worthy route to explore in plant breeding. Ultimately, it is likely that less recalcitrant biomass will allow substantial economic and resource savings in transport and in downstream biomass processing.

## Conclusions

Our analysis of miscanthus biomass revealed that the cell wall makeup and biomass quality are significantly distinct between plant organs, groups of genotypes and species. We identified a set of cell wall variables, including molecular features that affect the fine structure of the cell wall, that are distinct between genotypes with high or low saccharification efficiency performances and can therefore be used as markers to help interrogate future datasets. Specifically in stems, lignin has a predominant effect on recalcitrance, and its modification may therefore have a beneficial impact on saccharification. In foliar biomass, the picture is more complex as cell wall traits related to the nature and ornamentation of matrix glycans have more determinant positive or negative effects on saccharification and hence may be targets to improve the biorefining potential of leaves. Organ-derived differences in cell wall deconstruction will have distinct impacts on biomass valorisation, especially when considering the variation in leaf and stem contribution to total biomass across different miscanthus genotypes [24]. Thus, the development of miscanthus varieties with modified leaf-to-stem ratios may in itself lead to biorefining improvements.

Novel biomass processing methodologies are being developed to produce added-value chemical commodities from hemicellulose sugars, for instance, by converting xylose into xylitol [104–106]. Reports have also shown that many phenols of interest for nutraceutical, cosmetic and pharmaceutical industries occur in miscanthus biomass [107, 108]. This underlines the potential of miscanthus biomass valorisation through a cascading biorefining concept. Although miscanthus species are essentially undomesticated, in temperate climates they outperform many other C_4_ crops [109]. Furthermore, there is huge potential of their genetic resources to be utilised for crop improvement [110]. The results obtained in this study reinforce the value of a detailed exploration of such genotypic diversity in cell wall-related molecular traits to define and refine markers that can be exploited for the development of miscanthus as a renewable biorefinery feedstock. Our findings promote the idea that a holistic outlook of the cell wall is indispensable to improve biomass quality. Hence, instead of defining a single miscanthus biorefining ideotype, the development of a collection of varieties, taking into account target products, provides a more realistic and valuable approach.

## Additional files

**Additional file 1.** Listing of all plant cell wall glycan-directed monoclonal antibodies used in the glycome profiling screening.

**Additional file 2.** Full dataset of the immunofluorescent labelling study of glycan epitopes in transverse sections from miscanthus leaf and stem. The first panel refers to sections stained with toluidine blue.

**Additional file 3.** All measured values for the cell wall traits for each of eight miscanthus genotypes used in this study.

**Additional file 4.** ANOVA tables of results from enzymatic saccharification efficiency.

**Additional file 5.** Complete glycome profile of cell wall material from all samples used in this study.

**Additional file 6.** Correlation matrices between all measured cell wall compositional features and enzymatic saccharification efficiencies of glucose, xylose, arabinose.

**Additional file 7.** Box and whisker plots showing the distribution of biomass compositional measurements, and the *P*-values of *t* tests used to discriminate between genotypes with high or low saccharification efficiency indices.
