# Supplementary material for: Desirable plant cell wall traits for higher-quality miscanthus lignocellulosic biomass
Source: Biotechnol Biofuels. 2019 Apr 15;12:85. doi: 10.1186/s13068-019-1426-7 (PMC6463665; doi:10.1186/s13068-019-1426-7)
Supplement: Supplementary file 1 — Additional file 1. Listing of all plant cell wall glycan-directed monoclonal antibodies used in the glycome profiling screening. [file 13068_2019_1426_MOESM1_ESM.pdf]

Additional file 1

Listing of plant cell wall glycan-directed monoclonal antibodies (mAbs) used in the glycome profiling screening. The groupings of antibodies are based on a hierarchical clustering of ELISA data generated from a screen of all mAbs against a panel of plant polysaccharide preparations (Pattathil *et al.*, 2010), which grouped the mAbs per the predominant polysaccharides recognised. Most listed items contain a web link to the WallMabDB plant cell wall monoclonal antibody database (http://www.wallmabdb.net), which provides detailed descriptions of each mAb, including immunogen, antibody isotype, epitope structure (to the current known extent), supplier information, and related literature citations.

mAb subclass – based on Pattathil et al. 2010	mAb Name
Non-Fucosylated Xyloglucan-1	CCRC-M95
	CCRC-M101
Non-Fucosylated Xyloglucan-2	CCRC-M104
	CCRC-M89
	CCRC-M93
	CCRC-M87
	CCRC-M88
Non-Fucosylated Xyloglucan-3	CCRC-M100
	CCRC-M103
Non-Fucosylated Xyloglucan-4	CCRC-M58
	CCRC-M86
	CCRC-M55
	CCRC-M52
	CCRC-M99
Non-Fucosylated Xyloglucan-5	CCRC-M54
	CCRC-M48
	CCRC-M49
	CCRC-M96
	CCRC-M50
	CCRC-M51
	CCRC-M53
Non-Fucosylated Xyloglucan-6	CCRC-M57
Fucosylated Xyloglucan	CCRC-M102
	CCRC-M39
	CCRC-M106
	CCRC-M84
	CCRC-M1
Xylan-1/XG	CCRC-M111
	CCRC-M108
	CCRC-M109
Xylan-2	CCRC-M119
	CCRC-M115
	CCRC-M110
	CCRC-M105
Xylan-3	CCRC-M117
	CCRC-M113
	CCRC-M120
	CCRC-M118
	CCRC-M116
	CCRC-M114
Xylan-4	CCRC-M154
	CCRC-M150

mAb subclass – based on Pattathil et al. 2010	mAbNames
Xylan-5	CCRC-M144
	CCRC-M146
	CCRC-M145
	CCRC-M155
Xylan-6	CCRC-M153
	CCRC-M151
	CCRC-M148
	CCRC-M140
	CCRC-M139
	CCRC-M138
Xylan-7	CCRC-M160
	CCRC-M137
	CCRC-M152
	CCRC-M149
Galactomannan-1	CCRC-M75
	CCRC-M70
	CCRC-M74
Galactomannan-2	CCRC-M166
	CCRC-M168
	CCRC-M174
	CCRC-M175
Glucomannan	CCRC-M169
	CCRC-M170
β-Glucan	LAMP
	BG1
HG Backbone-1	CCRC-M131
	CCRC-M38
	JIM5
HG Backbone-2	JIM136
	JIM7
RG-I Backbone	CCRC-M69
	CCRC-M35
	CCRC-M36
	CCRC-M14
	CCRC-M129
	CCRC-M72
Linseed Mucilage RG-I	JIM3
	CCRC-M40
	CCRC-M161
	CCRC-M164
Physcomitrella Pectin	CCRC-M98
	CCRC-M94
RG-Ia	CCRC-M5
	CCRC-M2
RG-Ib	JIM137
	JIM101
	CCRC-M61
	CCRC-M30
RG-Ic	CCRC-M23
	CCRC-M17
	CCRC-M19
	CCRC-M18
	CCRC-M56
	CCRC-M16

mAb subclass – based on Pattathil et al. 2010	mAbNames
RG-I/Arabinogalactan	CCRC-M60
	CCRC-M41
	CCRC-M80
	CCRC-M79
	CCRC-M44
	CCRC-M33
	CCRC-M32
	CCRC-M13
	CCRC-M42
	CCRC-M24
	CCRC-M12
	CCRC-M7
	CCRC-M77
	CCRC-M25
	CCRC-M9
	CCRC-M128
	CCRC-M126
	CCRC-M134
	CCRC-M125
	CCRC-M123
	CCRC-M122
	CCRC-M121
	CCRC-M112
	CCRC-M21
	JIM131
	CCRC-M22
	JIM132
	JIM1
	CCRC-M15
	CCRC-M8
	JIM16
Arabinogalactan-1	JIM93
	JIM94
	JIM11
	MAC204
	JIM20
Arabinogalactan-2	JIM14
	JIM19
	JIM12
	CCRC-M133
	CCRC-M107
Arabinogalactan-3	JIM4
	CCRC-M31
	JIM17
	CCRC-M26
	JIM15
	JIM8
	CCRC-M85
	CCRC-M81
	MAC266
	PN16.4B4
Arabinogalactan-4	MAC207
	JIM133
	JIM13
	CCRC-M92
	CCRC-M91
	CCRC-M78
Unidentified	MAC265
	CCRC-M97
