# Supplementary material for: Desirable plant cell wall traits for higher-quality miscanthus lignocellulosic biomass
Source: Biotechnol Biofuels. 2019 Apr 15;12:85. doi: 10.1186/s13068-019-1426-7 (PMC6463665; doi:10.1186/s13068-019-1426-7)

Additional file 2

Cell wall glycan-directed mAbs used in the study of *in situ* immunolabelling of leaf and stem tissues from 8 miscanthus genotypes.

**mAb**	**mAb subclass – based on Pattathil *et al.* (2010)**
CCRC-M95	Non-fucosylated xyloglucan-1
CCRC-M87	Non-fucosylated xyloglucan-2
CCRC-M1	Fucosylated xyloglucan
CCRC-M114[BT]	Xylan-3
CCRC-M150	Xylan-4
CCRC-M154	Xylan-4
CCRC-M144[BT]	Xylan-5
CCRC-M155[BT]	Xylan-5
CCRC-M160	Xylan-7
CCRC-M137	Xylan-7
LAMP	(1→3)-β-glucan
BG1	Mixed-linkage (1→3, 1→4)-β-glucan
JIM7	Homogalacturonan backbone-2 (heavily esterified)
JIM5	Homogalacturonan backbone-1 (partially esterified and un-esterified)
CCRC-M38[BT]	Homogalacturonan backbone-1 (fully un-esterified)
CCRC-M14	Rhamnogalacturonan-I backbone
CCRC-M164	Epitopes akin to linseed mucilage rhamnogalacturonan-I
JIM137	Rhamnogalacturonan-Ib
CCRC-M7	RG-I/AGN (arabinogalactan side chains of rhamnogalacturonan-I)
CCRC-M12	RG-I/AGN (arabinogalactan side chains of rhamnogalacturonan-I)
CCRC-M133	Arabinogalactan-2
JIM13	Arabinogalactan-4 (arabinogalactan and arabinogalactan proteins)

[BT] mAbs used in combination with a 0.1M KOH base treatment.

Figure A

Transverse sections stained with toluidine blue of the eight miscanthus genotypes used for in situ immunolabelling. Leaf tissue of each genotype is in the left column and stem is in the right. Scale bar: 100µm.

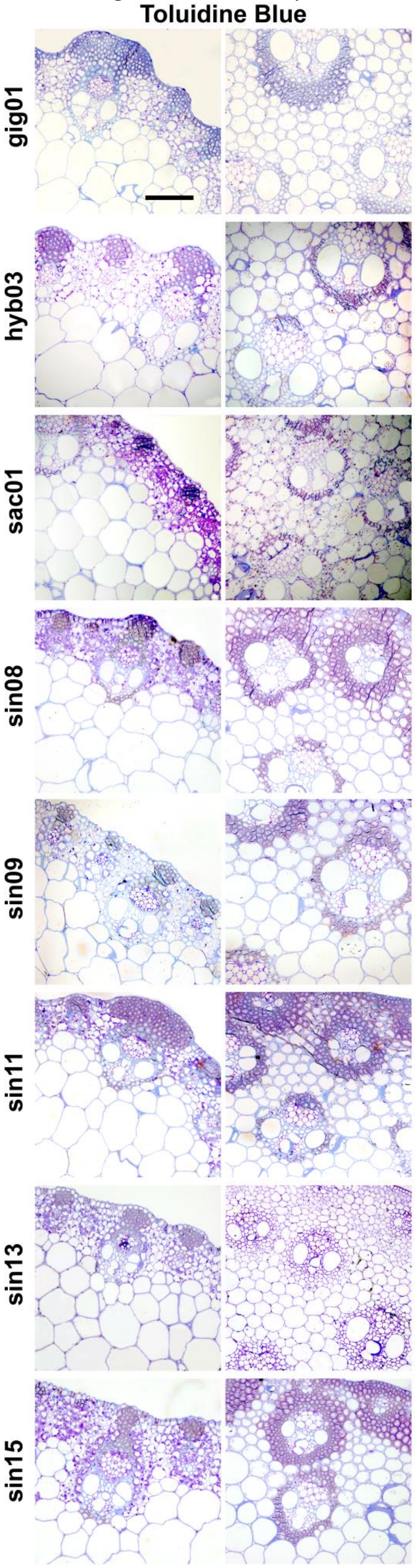

Figure B
Immunofluorescent labelling of transverse sections from leaves and stems of eight miscanthus genotypes with xyloglucan epitope binding mAbs. For each mAb, leaf is in the left column and stem is in the right. Scale bar: 100μm.

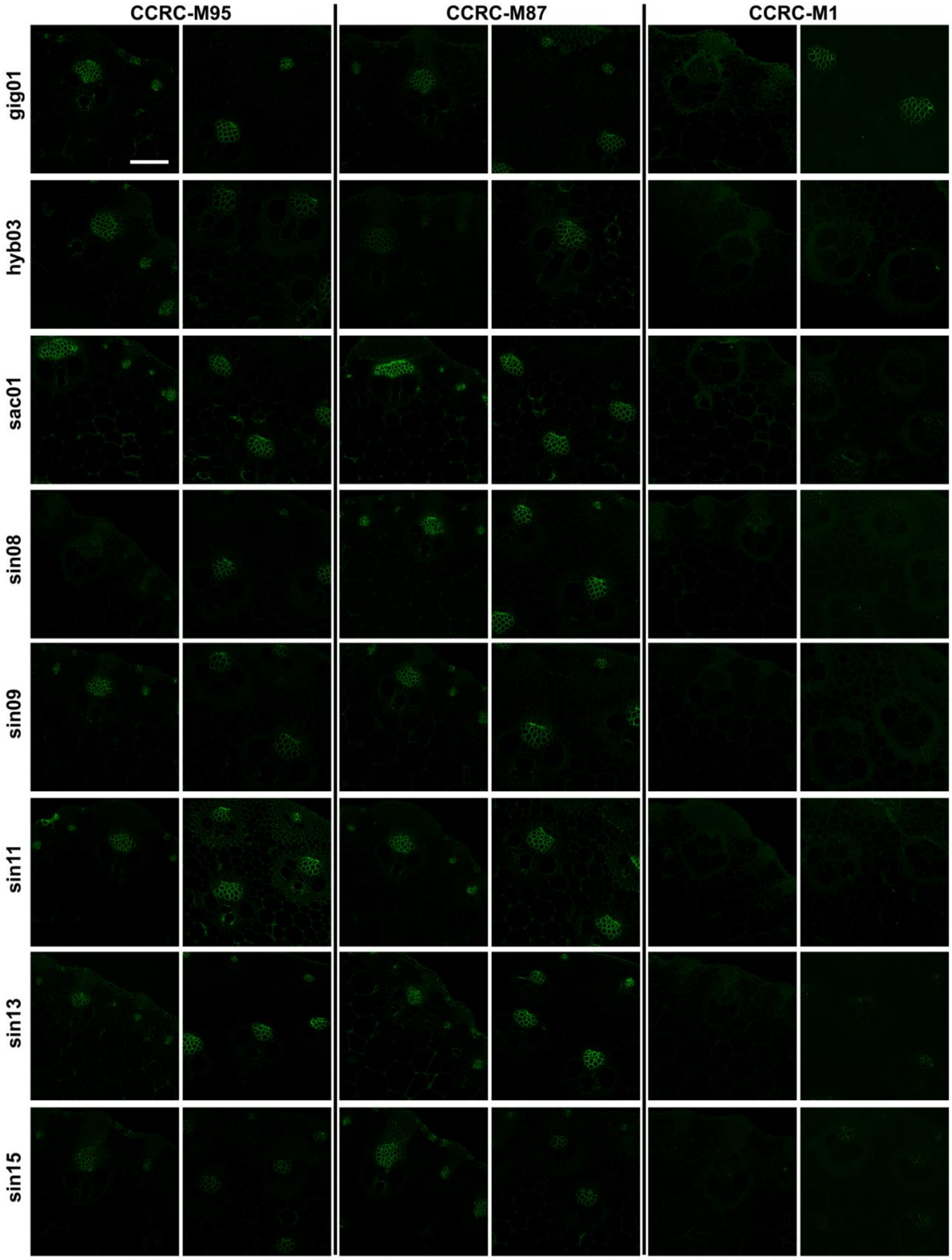

Figure C
Immunofluorescent labelling of transverse sections from leaves and stems of eight miscanthus genotypes with xylan epitope binding mAbs. For each mAb, leaf is in the left column and stem is in the right. Scale bar: 100µm.

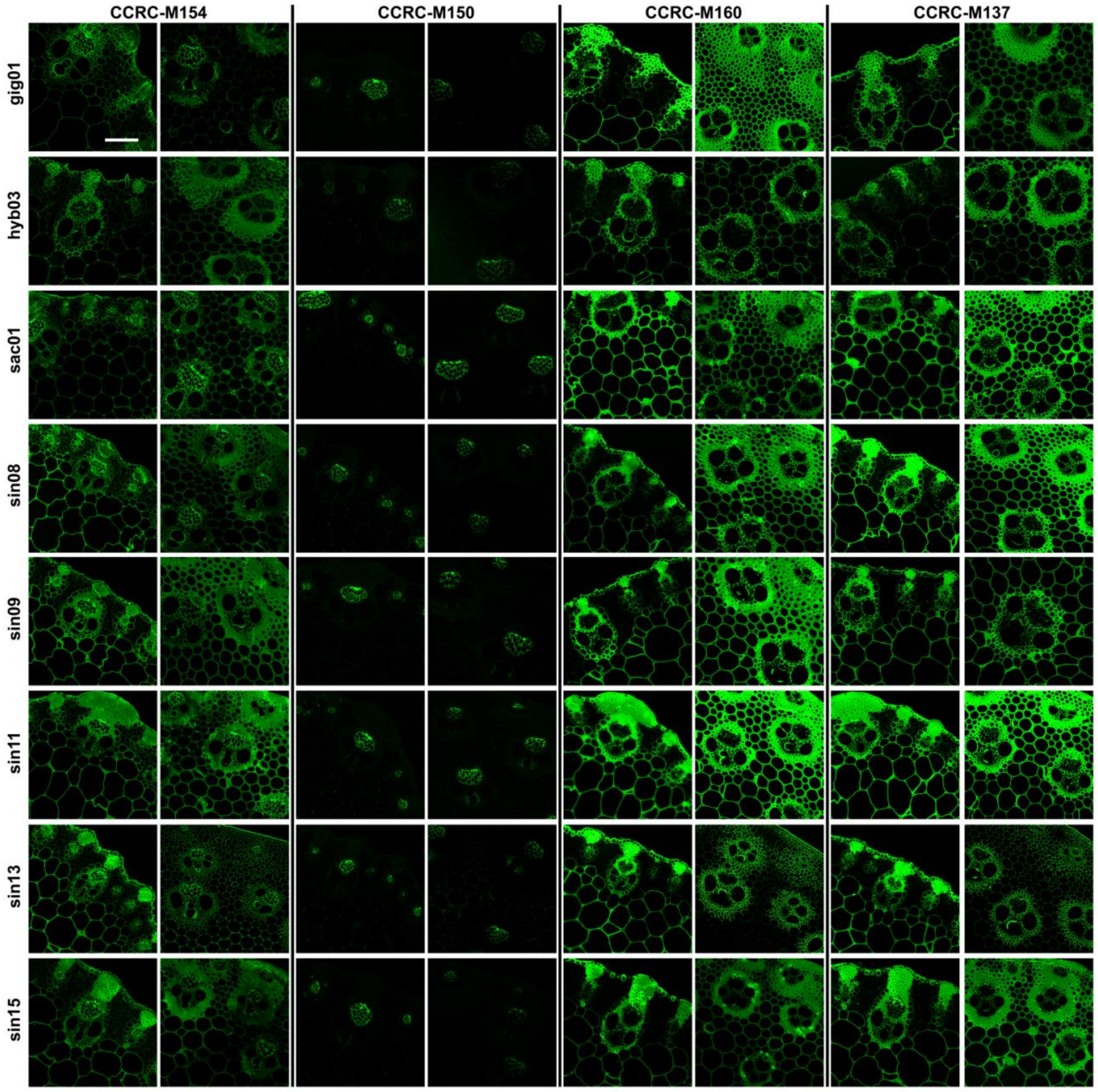

Figure D

Immunofluorescent labelling of transverse sections from leaves and stems of eight miscanthus genotypes with xylan epitope binding mAbs, before a base treatment with 0.1M KOH (controls). For each mAb, leaf is in the left column and stem is in the right. Scale bar: 100µm.

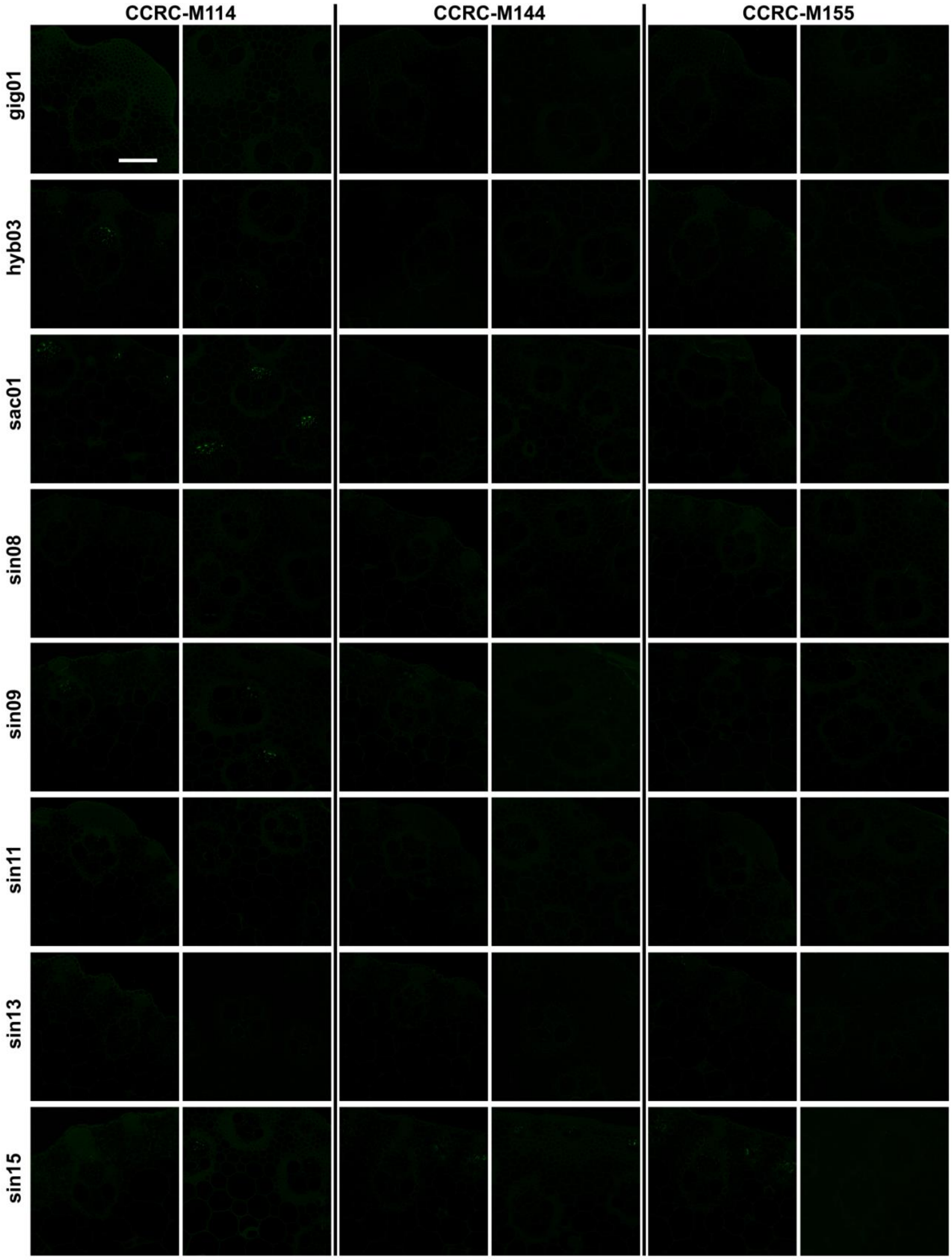

Figure E
Immunofluorescent labelling of transverse sections from leaves and stems of eight miscanthus genotypes with xylan epitope binding mAbs, after a base treatment (BT) with 0.1M KOH. For each mAb, leaf is in the left column and stem is in the right. Scale bar: 100μm.

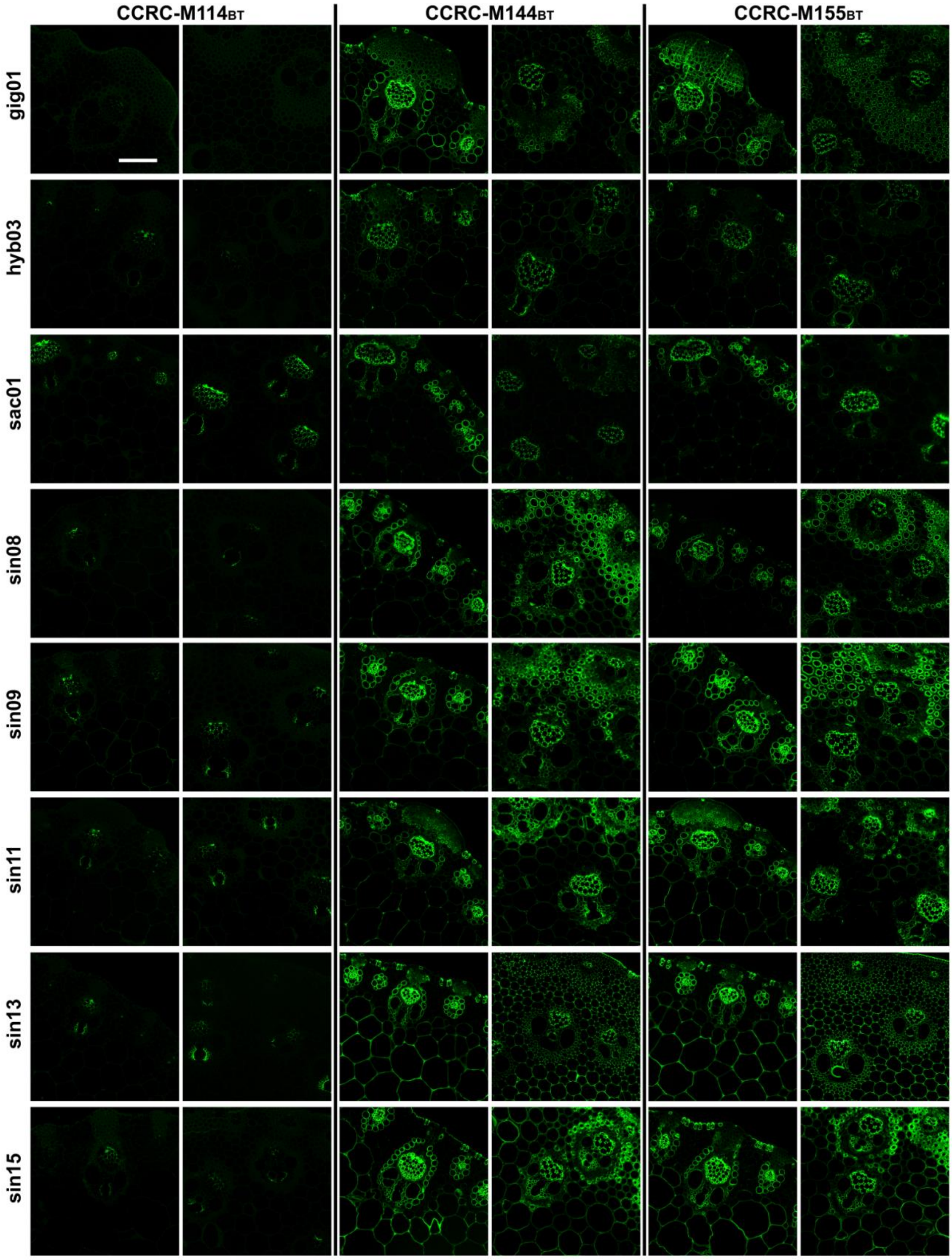

Figure F

Immunofluorescent labelling of transverse sections from leaves and stems of eight miscanthus genotypes with β-glucan epitope binding mAbs. For each mAb, leaf is in the left column and stem is in the right. Scale bar: 100µm.

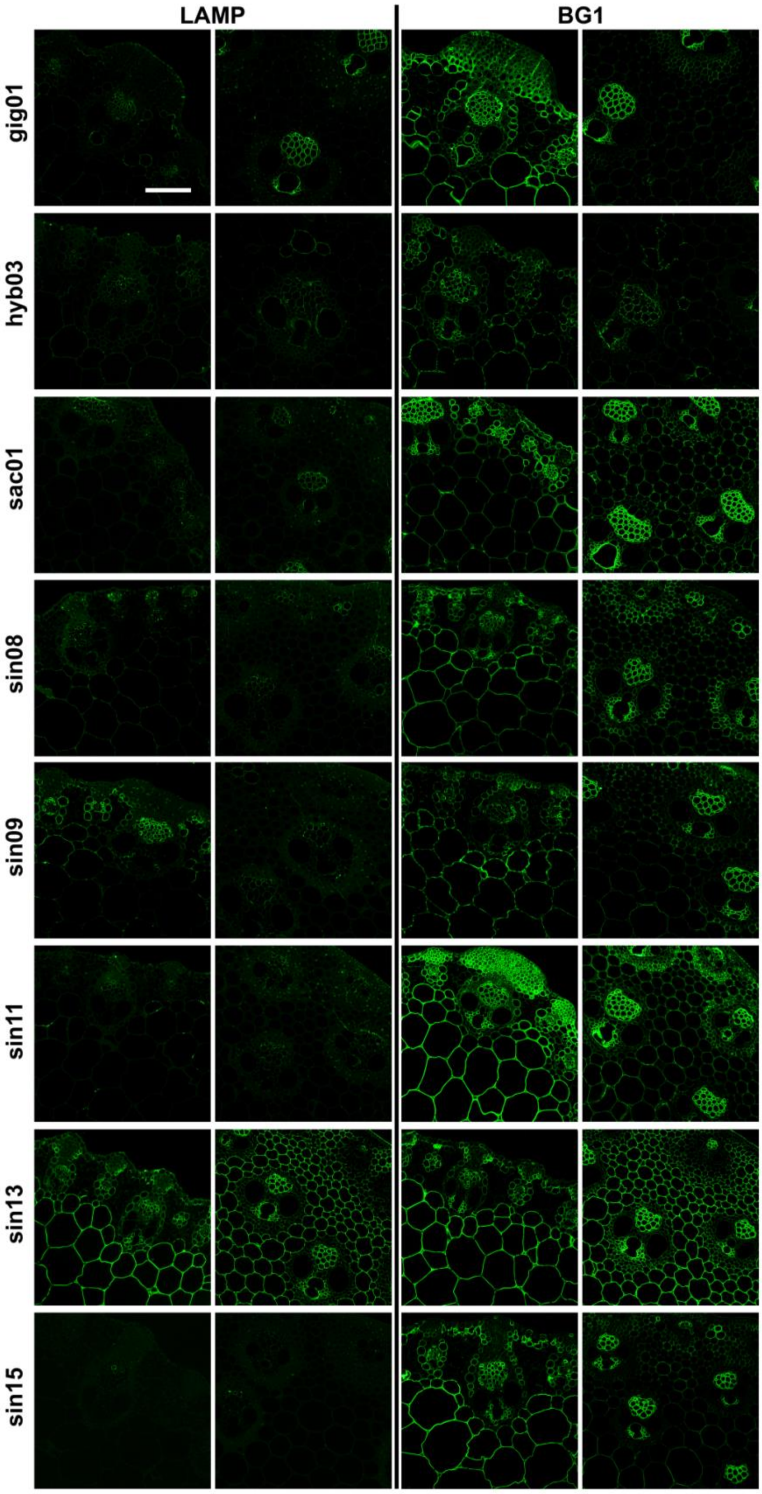

Figure G

Immunofluorescent labelling of transverse sections from leaves and stems of eight miscanthus genotypes with homogalacturonan epitope binding mAbs. For each mAb, leaf is in the left column and stem is in the right. For CCRC-M38 immunolabelling was performed before and after a base treatment (BT) with 0.1M KOH. Scale bar: 100µm.

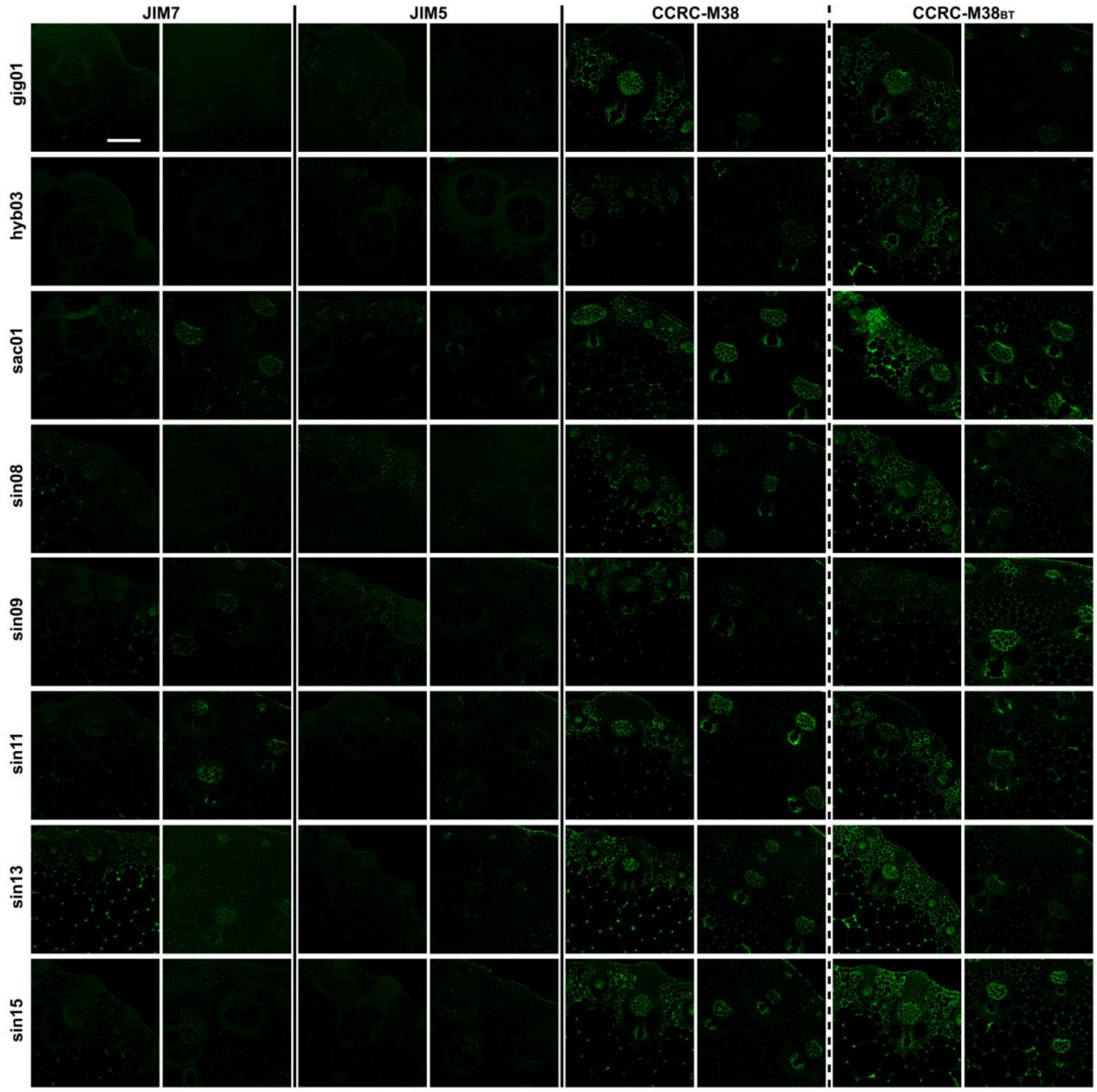

Figure H
Immunofluorescent labelling of transverse sections from leaves and stems of eight miscanthus genotypes with rhamnogalacturonan-I epitope binding mAbs. Scale bar: 100μm.

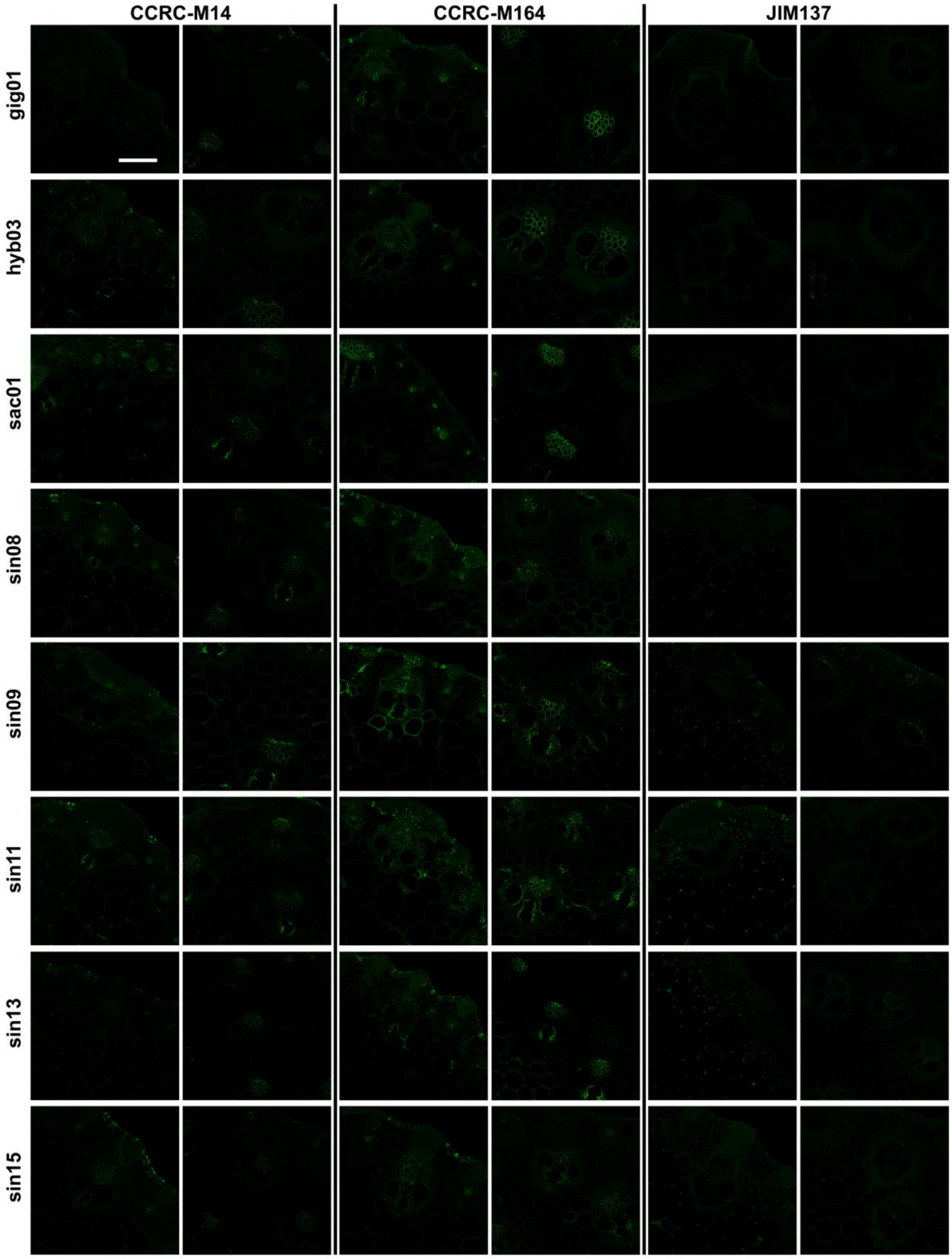

Figure I

Immunofluorescent labelling of transverse sections from leaves and stems of eight miscanthus genotypes with arabinogalactan epitope binding mAbs. For each mAb, leaf is in the left column and stem is in the right. Scale bar: 100µm.

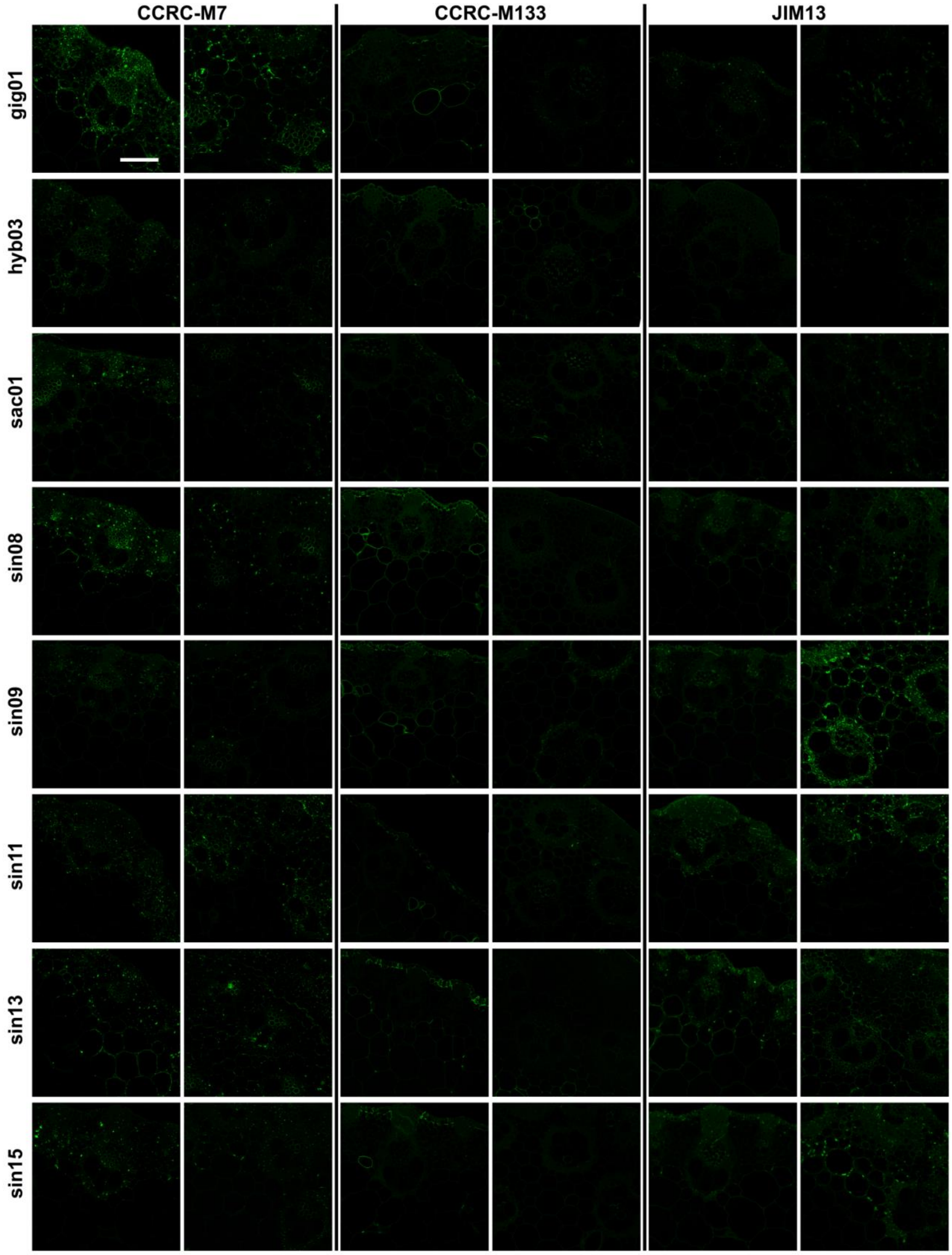

Figure J
Immunofluorescent labelling of transverse sections from leaves and stems of genotypes sac01, sin08, sin09 and sin11 with CCRC-M12, an arabinogalactan epitope binding mAb. Leaf sections are on the left column and stem sections are on the right. Scale bar: 100µm.

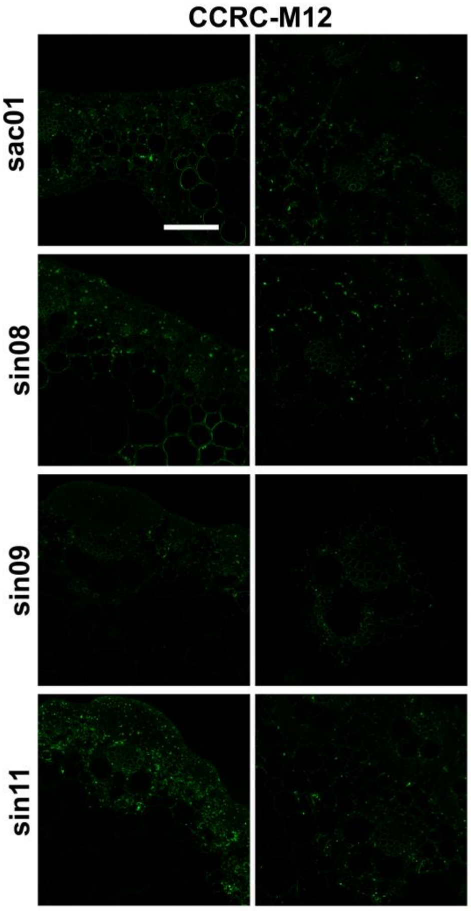

Supplement: Supplementary file 2 — Additional file 2. Full dataset of the immunofluorescent labelling study of glycan epitopes in transverse sections from miscanthus leaf and stem. The first panel refers to sections stained with toluidine blue. [file 13068_2019_1426_MOESM2_ESM.pdf]
