# Supplementary material for: Desirable plant cell wall traits for higher-quality miscanthus lignocellulosic biomass
Source: Biotechnol Biofuels. 2019 Apr 15;12:85. doi: 10.1186/s13068-019-1426-7 (PMC6463665; doi:10.1186/s13068-019-1426-7)
Supplement: Supplementary file 3 — Additional file 3. All measured values for the cell wall traits for each of eight miscanthus genotypes used in this study. [file 13068_2019_1426_MOESM3_ESM.pdf]

Additional file 3

All measured cell wall traits for each of the 8 miscanthus genotypes used in this study. Each value is the mean of triplicate biomass samples from mature plants, at peak biomass or senescence. N is a normalised value representing the total epitope abundance for specific mAbs (see main text for details).

	Peak Biomass															
	Leaf								Stem							
	gig01	hyb03	sac01	sin08	sin09	sin11	sin13	sin15	gig01	hyb03	sac01	sin08	sin09	sin11	sin13	sin15
**Glc (% CWM)**	42.85	40.90	47.84	35.52	40.44	39.66	37.42	38.36	49.04	48.96	54.48	41.11	45.57	43.47	42.10	43.93
**Xyl (% CWM)**	14.64	13.56	16.28	12.32	12.02	12.22	11.22	13.87	14.66	14.99	19.96	12.93	13.65	16.26	12.59	16.40
**%DW lignin**	19.87	19.07	17.85	21.07	20.17	20.38	19.47	21.17	22.08	24.12	20.73	21.10	22.58	22.15	16.03	21.66
**FA (% CWM)**	0.44	0.49	0.41	0.21	0.24	0.22	0.33	0.21	0.43	0.59	0.47	0.22	0.29	0.23	0.28	0.25
**pCA (% CWM)**	0.87	1.00	0.69	0.42	0.49	0.52	0.78	0.50	1.88	2.26	1.34	0.52	0.55	0.81	0.99	0.77
**Ara/Xyl**	0.17	0.17	0.15	0.20	0.18	0.29	0.18	0.22	0.07	0.07	0.06	0.11	0.08	0.11	0.12	0.11
**Acetate (% CWM)**	3.42	2.86	2.77	3.74	3.69	3.61	3.24	3.56	4.28	4.20	4.47	4.77	4.71	5.22	5.17	4.81
**N CCRC-M87 (OD/g CWM)**	4960.6	4841.7	5920.9	7186.6	5959.4	9414.7	4838.2	5315.7	4887.1	3440.1	4327.7	4419.2	6384.2	4976.3	6391.2	3258.0
**N CCRC-M154 (OD/g CWM)**	10215.5	9925.3	12197.0	15300.2	12382.1	16010.0	12658.4	12684.5	7038.7	7006.0	6504.1	8411.7	8097.1	8789.7	9176.4	7392.1
**N CCRC-M144 (OD/g CWM)**	12028.1	11870.2	14067.9	17522.6	13897.9	18631.5	15736.7	15742.7	8507.5	9249.0	8406.5	11147.1	9423.7	10833.1	11455.6	9371.4
**N CCRC-M137 (OD/g CWM)**	9829.9	9440.6	11909.6	15210.5	12435.9	15509.6	12453.2	12624.4	7009.7	7524.6	6974.9	9634.1	8675.0	9169.0	9243.6	7968.0
**N BG1 (OD/g CWM)**	10833.9	9693.2	11914.8	8584.8	11720.7	14841.8	7442.0	6905.0	5808.0	5068.4	6163.5	3839.7	7606.9	8314.6	6407.4	3485.2
**N CCRC-M7 (OD/g CWM)**	4252.9	4205.6	6862.8	5242.6	5660.7	8972.7	6538.8	5597.9	1895.3	1673.2	2189.1	1464.6	1773.4	1657.2	2856.3	941.7
**N CCRC-M38 (OD/g CWM)**	5937.4	6213.5	7044.5	8840.7	8124.2	11439.6	9489.4	8723.0	4430.5	4180.5	4290.6	4667.4	5132.4	4975.0	6598.3	4098.4
**GlcE (% total Glc)**	20.49	19.10	18.85	17.60	23.15	22.61	16.50	15.28	16.82	14.55	17.33	18.94	14.58	16.69	28.02	17.49
**GlcE rank**	High	High	High	Low	High	High	Low	Low	High	Low	High	High	Low	High	High	High
**XylE (% total Xyl)**	5.18	4.57	4.90	5.24	8.59	4.53	6.08	3.21	14.52	12.89	11.26	17.51	13.72	11.31	18.26	15.28
**XylE rank**	High	Low	High	High	High	Low	High	Low	High	High	Low	High	High	Low	High	High
**AraE (% total Ara)**	6.81	5.56	7.85	4.67	7.32	3.68	5.13	3.60	9.50	8.52	8.97	6.58	9.01	5.71	13.97	7.56

	Senescence															
	Leaf								Stem							
	gig01	hyb03	sac01	sin08	sin09	sin11	sin13	sin15	gig01	hyb03	sac01	sin08	sin09	sin11	sin13	sin15
**Glc (% CWM)**	44.83	42.92	45.45	39.24	42.66	42.68	35.50	41.47	50.14	47.31	54.99	41.03	43.35	42.05	39.45	41.84
**Xyl (% CWM)**	14.78	17.03	17.43	13.63	16.73	17.22	11.03	15.02	15.33	15.35	20.16	13.31	15.02	15.61	12.69	15.29
**%DW lignin**	20.50	19.95	21.10	22.51	23.96	24.47	22.33	22.90	23.19	23.70	23.85	23.88	24.33	25.70	22.33	24.99
**FA (% CWM)**	0.49	0.49	0.33	0.19	0.24	0.20	0.25	0.20	0.38	0.52	0.44	0.22	0.22	0.22	0.23	0.33
**pCA (% CWM)**	1.13	1.11	0.70	0.51	0.54	0.62	0.64	0.52	1.67	1.69	1.52	0.65	0.67	0.90	0.99	0.72
**Ara/Xyl**	0.16	0.14	0.14	0.15	0.13	0.19	0.22	0.16	0.07	0.07	0.07	0.10	0.09	0.10	0.10	0.10
**Acetate (% CWM)**	4.08	3.35	3.12	5.05	4.94	4.62	3.91	4.48	4.10	3.96	4.09	4.78	5.23	5.09	5.13	4.90
**N CCRC-M87 (OD/g CWM)**	6905.7	6343.9	4885.4	6028.8	4820.3	6195.4	8484.2	4865.5	3979.1	4937.1	3961.6	4688.6	6214.6	3942.2	6095.5	4581.5
**N CCRC-M154 (OD/g CWM)**	10959.6	11858.6	12211.6	13007.5	12943.0	13409.1	12457.6	13062.3	6405.8	10206.2	8101.8	7834.3	10012.9	7404.5	9714.0	10045.3
**N CCRC-M144 (OD/g CWM)**	13084.5	14925.2	13861.7	16626.3	14500.1	15634.1	16105.4	15815.4	7843.7	12261.0	9304.5	9837.8	11479.3	8949.8	11367.1	12905.1
**N CCRC-M137 (OD/g CWM)**	10674.8	12068.0	11892.0	13063.4	13111.2	12923.5	12791.8	13126.8	6878.7	10753.6	8386.5	8643.5	10764.9	7650.1	9677.1	10487.4
**N BG1 (OD/g CWM)**	11023.4	12530.2	13536.4	9112.4	14012.9	7628.0	7693.0	7688.3	5442.5	6424.6	6141.9	4956.0	8311.2	4145.8	6602.2	4869.9
**N CCRC-M7 (OD/g CWM)**	4703.6	6730.7	6045.4	1546.4	1695.7	2288.7	5543.9	2041.7	1874.3	2824.8	2146.4	2506.3	2303.8	1619.6	3809.1	973.7
**N CCRC-M38 (OD/g CWM)**	7406.7	9308.2	7532.3	8762.1	7815.5	7634.5	9812.3	8084.1	3752.4	4320.7	3865.4	5528.3	5070.0	3636.4	7150.6	5749.4
**GlcE (% total Glc)**	19.81	21.46	14.16	12.95	12.79	14.19	19.08	12.37	13.27	16.07	18.16	11.44	9.76	10.27	16.22	10.61
**GlcE rank**	High	High	Low	Low	Low	Low	High	Low	Low	Low	High	Low	Low	Low	High	Low
**XylE (% total Xyl)**	5.53	5.70	2.68	4.14	2.70	3.52	5.10	3.89	10.81	13.40	16.16	8.73	7.30	7.35	12.67	7.62
**XylE rank**	High	High	Low	Low	Low	Low	High	Low	Low	High	High	Low	Low	Low	Low	Low
**AraE (% total Ara)**	6.70	8.68	4.00	3.05	3.27	2.41	5.52	3.29	9.14	10.06	6.84	3.53	4.92	3.86	8.84	3.60
