# Supplementary material for: Desirable plant cell wall traits for higher-quality miscanthus lignocellulosic biomass
Source: Biotechnol Biofuels. 2019 Apr 15;12:85. doi: 10.1186/s13068-019-1426-7 (PMC6463665; doi:10.1186/s13068-019-1426-7)
Supplement: Supplementary file 4 — Additional file 4. ANOVA tables of results from enzymatic saccharification efficiency. [file 13068_2019_1426_MOESM4_ESM.pdf]

Additional file 4

ANOVA tables of results from enzymatic saccharification efficiency.

Effect	Sum of squares	Degrees of freedom	Mean square	*F*-ratio	*P*-value	Effect size (η^2)
**GlcE All**						
Genotype	205.52	7	29.36	356.70	*<0.001*	0.199
Organ	56.93	1	56.93	691.70	*<0.001*	0.055
Developmental Stage	267.22	1	267.22	3246.40	*<0.001*	0.259
Genotype × Organ	201.04	7	28.72	348.90	*<0.001*	0.195
Genotype × Dev. Stage	146.40	7	20.91	254.10	*<0.001*	0.142
Organ × Dev. Stage	8.81	1	8.81	107.00	*<0.001*	0.009
Genotype × Organ × DS	143.66	7	20.52	249.30	*<0.001*	0.139
Error	2.63	32	0.08			
**Total**	1032.21	63				
**GlcE Leaf**						
Genotype	141.38	7	20.20	444.70	*<0.001*	0.367
Developmental Stage	89.50	1	89.50	1970.80	*<0.001*	0.232
Genotype × Dev. Stage	153.50	7	21.93	482.90	*<0.001*	0.399
Error	0.73	16	0.05			
**Total**	385.09	31				
**GlcE Stem**						
Genotype	265.18	7	37.88	317.78	*<0.001*	0.449
Developmental Stage	186.54	1	186.54	1564.73	*<0.001*	0.316
Genotype × Dev. Stage	136.56	7	19.51	163.65	*<0.001*	0.231
Error	1.91	16	0.12			
**Total**	590.18	31				
**XylE All**						
Genotype	75.64	7	10.81	79.44	*<0.001*	0.055
Organ	949.04	1	949.04	6977.50	*<0.001*	0.686
Developmental Stage	98.80	1	98.80	726.38	*<0.001*	0.071
Genotype × Organ	48.48	7	6.93	50.92	*<0.001*	0.035
Genotype × Dev. Stage	94.74	7	13.53	99.50	*<0.001*	0.068
Organ × Dev. Stage	29.39	1	29.39	216.10	*<0.001*	0.021
Genotype × Organ × DS	83.95	7	11.99	88.18	*<0.001*	0.061
Error	4.35	32	0.14			
**Total**	1384.40	63				
**XylE Leaf**						
Genotype	19.56	7	2.79	21.61	*<0.001*	0.295
Developmental Stage	10.21	1	10.21	78.92	*<0.001*	0.154
Genotype × Dev. Stage	34.48	7	4.93	38.09	*<0.001*	0.520
Error	2.07	16	0.13			
**Total**	66.32	31				
**XylE Stem**						
Genotype	104.56	7	14.94	104.67	*<0.001*	0.283
Developmental Stage	117.98	1	117.98	826.81	*<0.001*	0.320
Genotype × Dev. Stage	144.21	7	20.60	144.37	*<0.001*	0.391
Error	2.28	16	0.14			
**Total**	369.03	31				
**AraE All**						
Genotype	188.79	7	26.97	37.98	*<0.001*	0.407
Organ	95.43	1	95.43	134.39	*<0.001*	0.206
Developmental Stage	44.61	1	44.61	62.83	*<0.001*	0.096
Genotype × Organ	32.60	7	4.66	6.56	*<0.001*	0.070
Genotype × Dev. Stage	53.48	7	7.64	10.76	*<0.001*	0.115
Organ × Dev. Stage	8.03	1	8.03	11.31	*0.002*	0.017
Genotype × Organ × DS	17.90	7	2.56	3.60	*0.006*	0.039
Error	22.72	32	0.71			
**Total**	463.57	63				
**AraE Leaf**						
Genotype	64.35	7	9.19	42.57	*<0.001*	0.568
Developmental Stage	7.39	1	7.39	34.23	*<0.001*	0.065
Genotype × Dev. Stage	38.11	7	5.44	25.21	*<0.001*	0.336
Error	3.46	16	0.22			
**Total**	113.31	31				
**AraE Stem**						
Genotype	157.04	7	22.44	18.63	*<0.001*	0.616
Developmental Stage	45.26	1	45.26	37.58	*<0.001*	0.178
Genotype × Dev. Stage	33.27	7	4.75	3.95	*0.011*	0.131
Error	19.27	16	1.20			
**Total**	254.83	31				
