# Supplementary material for: Desirable plant cell wall traits for higher-quality miscanthus lignocellulosic biomass
Source: Biotechnol Biofuels. 2019 Apr 15;12:85. doi: 10.1186/s13068-019-1426-7 (PMC6463665; doi:10.1186/s13068-019-1426-7)

Additional file 5
Figure A

Glycome profile of cell wall material (CWM) from the leaves of miscanthus at the peak biomass stage (PBL). The CWM of eight miscanthus genotypes was sequentially extracted with ammonium oxalate (AO), sodium carbonate (CA), 1M KOH (1M), 4M KOH (4M) and delignified with sodium chlorite (CH) followed by another extraction with 4M KOH post-chlorite (PC). Corresponding genotypes and extracts are labelled below each profile. Each extract was probed against an array of plant glycan-directed monoclonal antibodies (panel on the right of the figure). Antibody binding strength depicted in function of optical density (OD) is presented as a colour gradient ranging from black (no binding) to yellow (OD=1.3). The bar graphs at the top indicate the amount of sugars recovered in the solubilised extracts per gram of CWM.

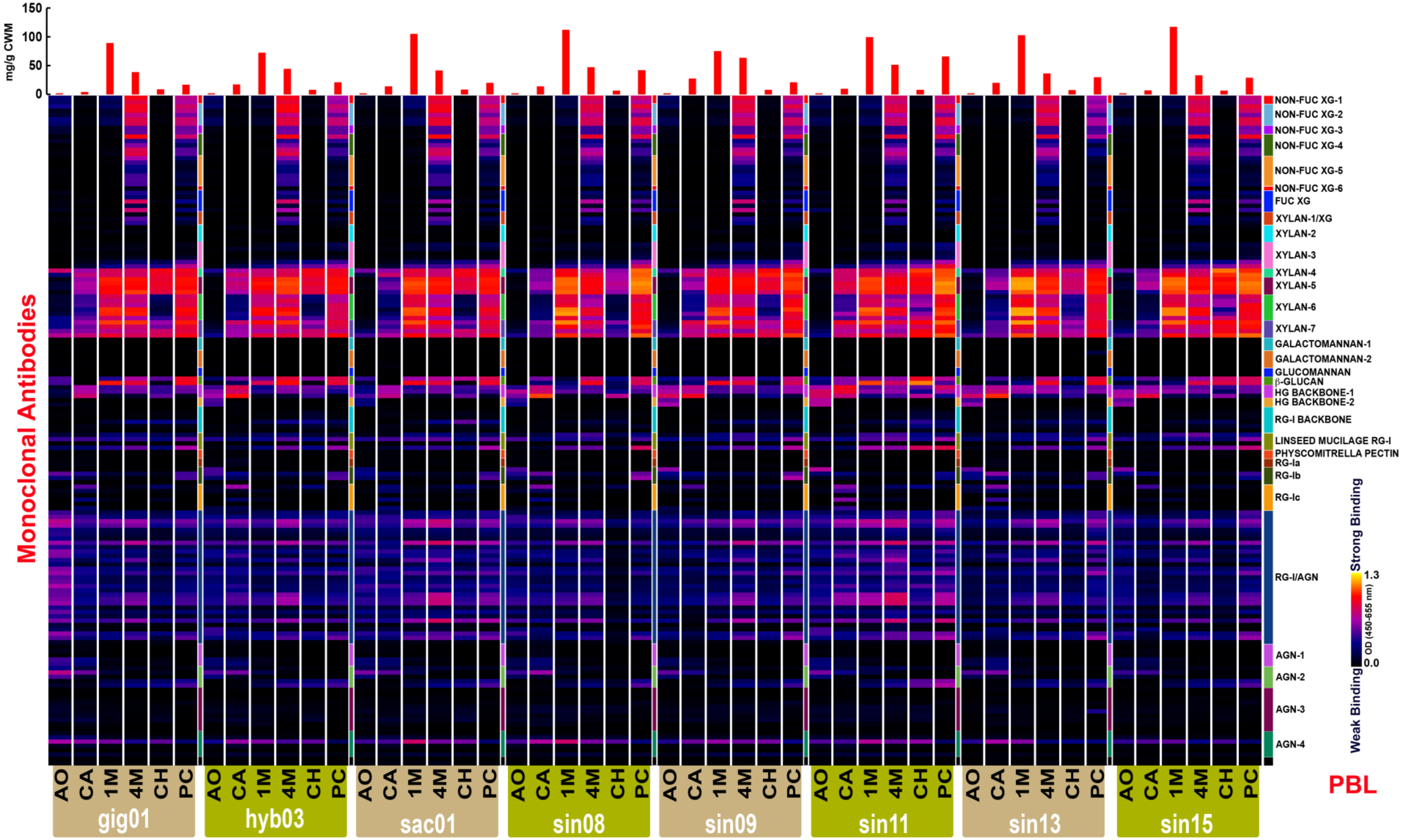

Figure B

Glycome profile of cell wall material (CWM) from the leaves of senesced miscanthus (SSL). The CWM of eight miscanthus genotypes was sequentially extracted with ammonium oxalate (AO), sodium carbonate (CA), 1M KOH (1M), 4M KOH (4M) and delignified with sodium chlorite (CH) followed by another extraction with 4M KOH post-chlorite (PC). Corresponding genotypes and extracts are labelled below each profile. Each extract was probed against an array of plant glycan-directed monoclonal antibodies (panel on the right of the figure). Antibody binding strength depicted in function of optical density (OD) is presented as a colour gradient ranging from black (no binding) to yellow (OD=1.3). The bar graphs at the top indicate the amount of sugars recovered in the solubilised extracts per gram of CWM.

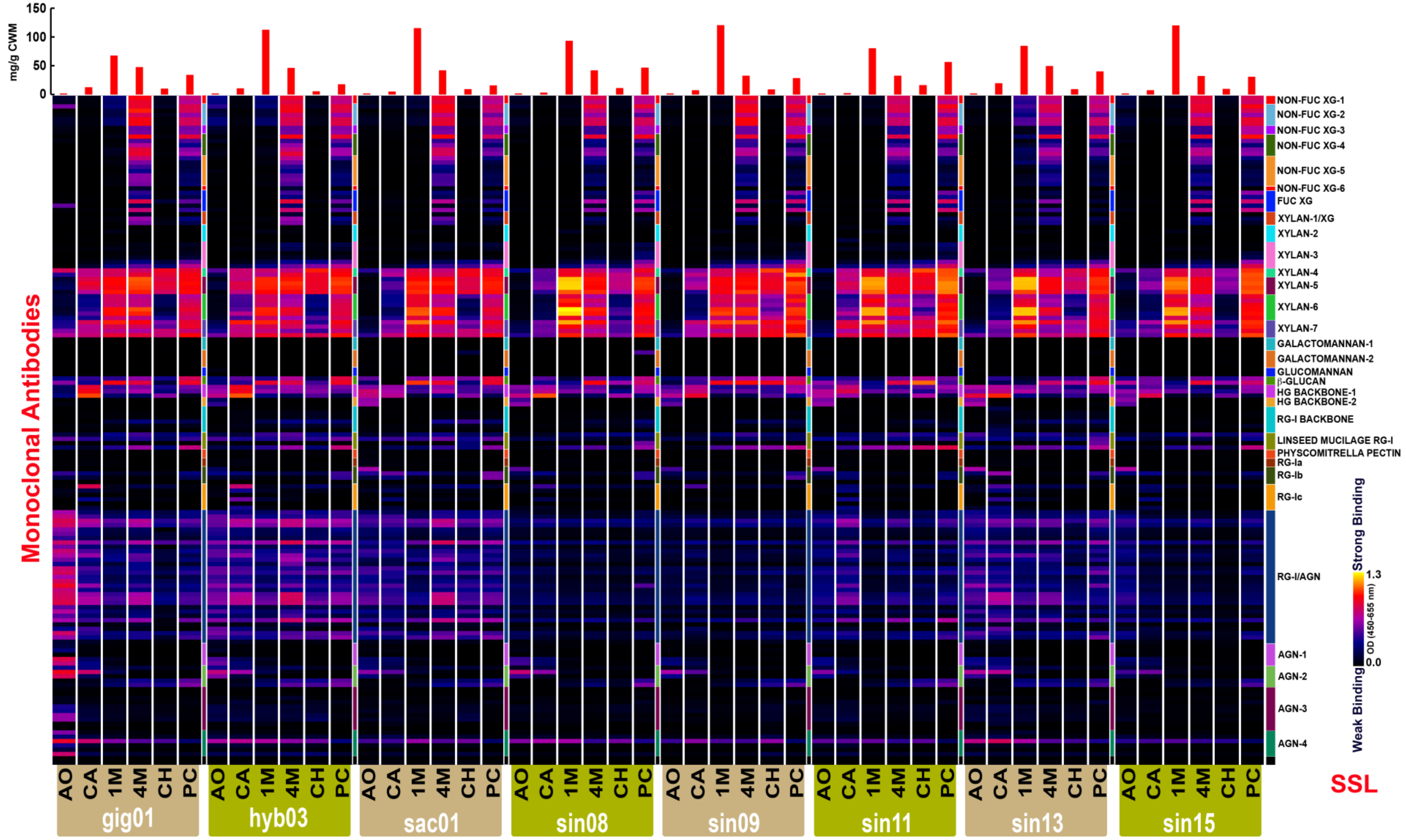

Figure C

Glycome profile of cell wall material (CWM) from the stem of miscanthus at the peak biomass stage (PBS). The CWM of eight miscanthus genotypes was sequentially extracted with ammonium oxalate (AO), sodium carbonate (CA), 1M KOH (1M), 4M KOH (4M) and delignified with sodium chlorite (CH) followed by another extraction with 4M KOH post-chlorite (PC). Corresponding genotypes and extracts are labelled below each profile. Each extract was probed against an array of plant glycan-directed monoclonal antibodies (panel on the right of the figure). Antibody binding strength depicted in function of optical density (OD) is presented as a colour gradient ranging from black (no binding) to yellow (OD=1.3). The bar graphs at the top indicate the amount of sugars recovered in the solubilised extracts per gram of CWM.

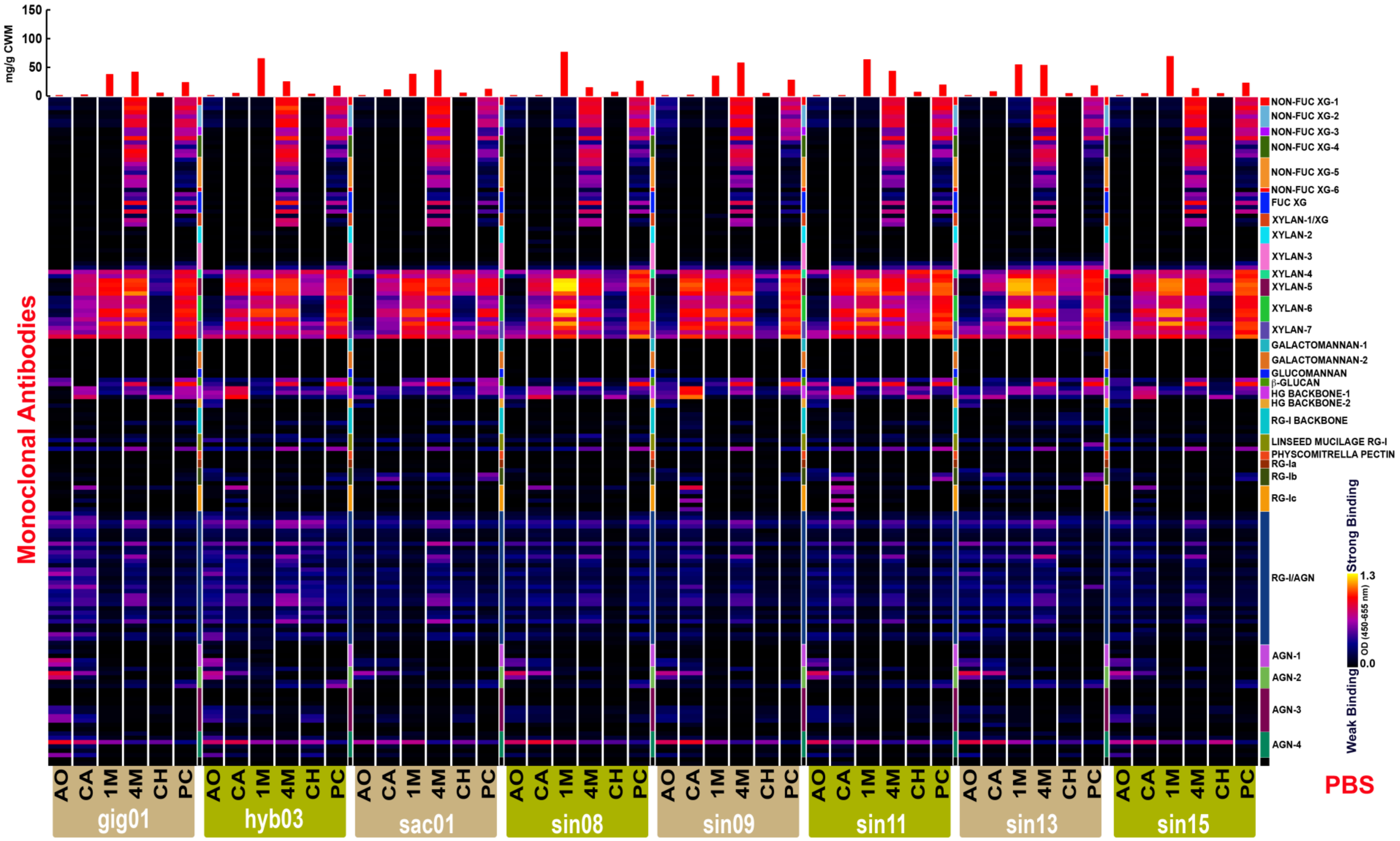

Figure D

Glycome profile of cell wall material (CWM) from the stem of senesced miscanthus (SSS). The CWM of eight miscanthus genotypes was sequentially extracted with ammonium oxalate (AO), sodium carbonate (CA), 1M KOH (1M), 4M KOH (4M) and delignified with sodium chlorite (CH) followed by another extraction with 4M KOH post-chlorite (PC). Corresponding genotypes and extracts are labelled below each profile. Each extract was probed against an array of plant glycan-directed monoclonal antibodies (panel on the right of the figure). Antibody binding strength depicted in function of optical density (OD) is presented as a colour gradient ranging from black (no binding) to yellow (OD=1.3). The bar graphs at the top indicate the amount of sugars recovered in the solubilised extracts per gram of CWM.

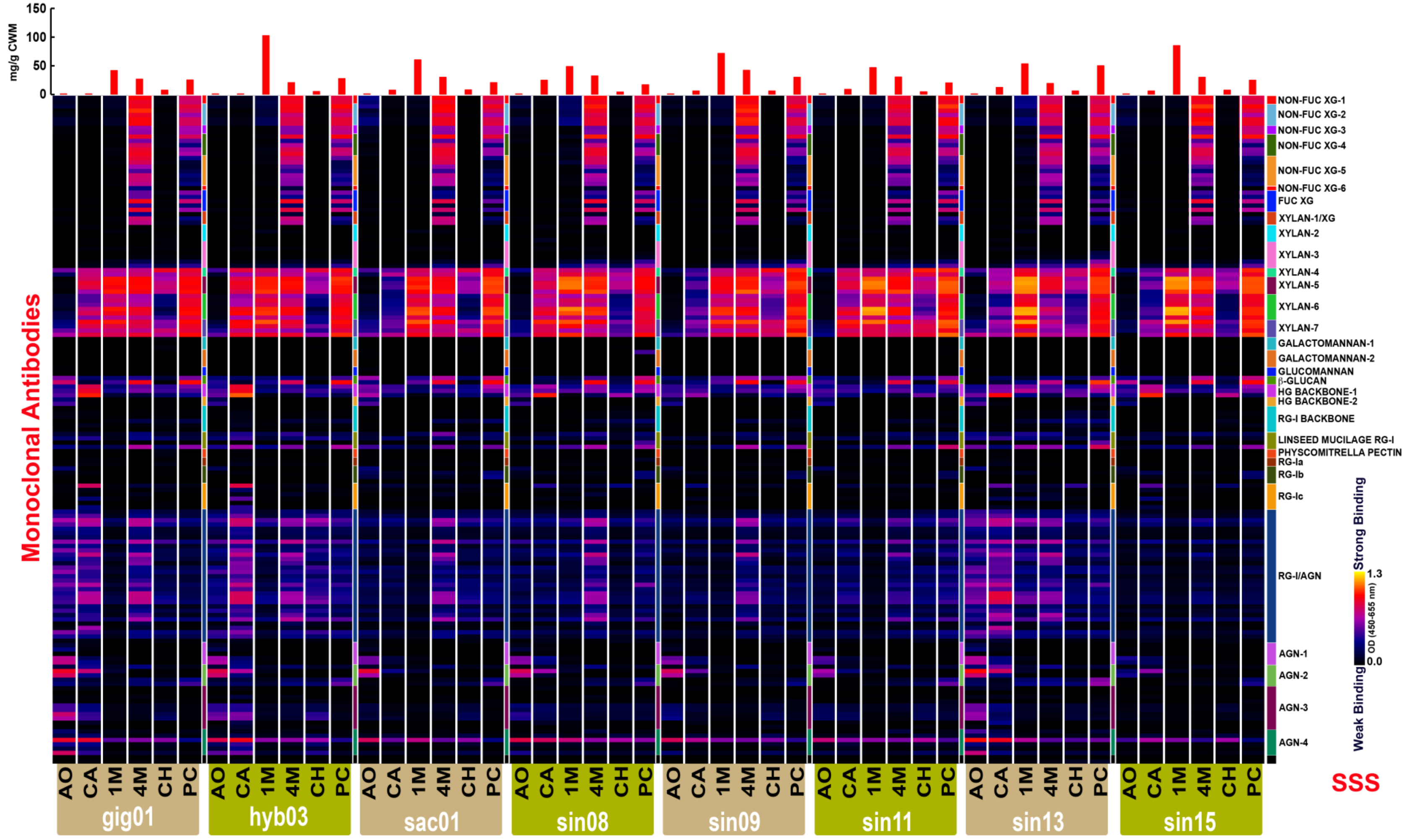

Supplement: Supplementary file 5 — Additional file 5. Complete glycome profile of cell wall material from all samples used in this study. [file 13068_2019_1426_MOESM5_ESM.pdf]
