# Supplementary material for: Desirable plant cell wall traits for higher-quality miscanthus lignocellulosic biomass
Source: Biotechnol Biofuels. 2019 Apr 15;12:85. doi: 10.1186/s13068-019-1426-7 (PMC6463665; doi:10.1186/s13068-019-1426-7)
Supplement: Supplementary file 6 — Additional file 6. Correlation matrices between all measured cell wall compositional features and enzymatic saccharification efficiencies of glucose, xylose, arabinose. [file 13068_2019_1426_MOESM6_ESM.pdf]

Additional file 6
Table A
For leaf biomass, correlation matrix between all measured cell wall compositional features and with enzymatic saccharification efficiencies of glucose (GlcE), xylose (XylE), arabinose (AraE). Pearson correlation coefficients values are presented, within a colour gradient ranging from red (-1) to green (1). *P*-values, for statistically significant correlations (α=0.05; n=32) are highlighted in red. N is a normalised value representing the total epitope abundance for specific mAbs (see main text for details).

	GlcE (% total Glc)	XylE (% total Xyl)	Glucose (% CWM)	Xylose (% CWM)	%DW lignin	Ferulic acid (ester-linked; % CWM)	p-Coumaric acid (ester-linked; % CWM)	Arabinose/Xylose	Acetate (% CWM)	AraE (% total Ara)	N M87 (OD/g CWM)	N M154 (OD/g CWM)	N M144 (OD/g CWM)	N M137 (OD/g CWM)	N BG1 (OD/g CWM)	N M7 (OD/g CWM)	N M38 (OD/g CWM)
**GlcE (% total Glc)**	1.00																

**XylE (% total Xyl)**	0.73	1.00															
	0.000	---															
**Glucose (% CWM)**	-0.01	-0.18	1.00														
	0.975	0.312	---														
**Xylose (% CWM)**	-0.36	-0.51	0.80	1.00													
	0.044	0.003	0.000	---													
**%DW lignin**	-0.65	-0.50	-0.21	0.22	1.00												
	0.000	0.004	0.254	0.234	---												
**Ferulic acid (ester-linked; % CWM)**	0.45	0.23	0.52	0.27	-0.64	1.00											
	0.009	0.212	0.002	0.129	0.000	---											
**p-Coumaric acid (ester-linked; % CWM)**	0.36	0.19	0.42	0.27	-0.43	0.93	1.00										
	0.042	0.299	0.016	0.139	0.015	0.000	---										
**Arabinose/Xylose**	0.36	0.09	-0.57	-0.62	-0.04	-0.39	-0.34	1.00									
	0.040	0.627	0.001	0.000	0.840	0.029	0.059	---									
**Acetate (% CWM)**	-0.54	-0.27	-0.17	0.10	0.87	-0.56	-0.36	-0.11	1.00								
	0.001	0.131	0.353	0.570	0.000	0.001	0.043	0.564	---								
**AraE (% total Ara)**	0.74	0.69	0.30	-0.02	-0.73	0.73	0.60	-0.24	-0.58	1.00							
	0.000	0.000	0.096	0.930	0.000	0.000	0.000	0.195	0.001	---							
**N M87 (OD/g CWM)**	0.50	0.21	-0.34	-0.41	0.02	-0.20	-0.14	0.72	0.06	0.04	1.00						
	0.004	0.258	0.061	0.021	0.916	0.268	0.460	0.000	0.730	0.829	---						
**N M154 (OD/g CWM)**	-0.12	-0.13	-0.41	-0.23	0.33	-0.72	-0.69	0.56	0.31	-0.49	0.56	1.00					
	0.519	0.481	0.019	0.197	0.062	0.000	0.000	0.001	0.089	0.004	0.001	---					
**N M144 (OD/g CWM)**	-0.16	-0.12	-0.57	-0.35	0.35	-0.71	-0.61	0.61	0.34	-0.48	0.60	0.93	1.00				
	0.395	0.503	0.001	0.051	0.052	0.000	0.000	0.000	0.054	0.005	0.000	0.000	---				
**N M137 (OD/g CWM)**	-0.14	-0.10	-0.46	-0.25	0.38	-0.75	-0.71	0.51	0.36	-0.46	0.55	0.99	0.94	1.00			
	0.442	0.575	0.008	0.166	0.033	0.000	0.000	0.003	0.042	0.008	0.001	0.000	0.000	---			
**N BG1 (OD/g CWM)**	0.36	-0.01	0.53	0.35	-0.23	0.28	0.16	-0.15	-0.16	0.26	0.16	0.06	-0.15	0.03	1.00		
	0.043	0.952	0.002	0.049	0.215	0.120	0.395	0.412	0.385	0.151	0.388	0.754	0.406	0.864	---		
**N M7 (OD/g CWM)**	0.70	0.36	-0.04	-0.30	-0.70	0.26	0.15	0.50	-0.75	0.47	0.47	0.20	0.18	0.16	0.34	1.00	
	0.000	0.040	0.848	0.097	0.000	0.148	0.400	0.003	0.000	0.007	0.007	0.270	0.322	0.381	0.058	---	
**N M38 (OD/g CWM)**	0.15	0.10	-0.56	-0.47	0.13	-0.47	-0.34	0.66	0.14	-0.21	0.68	0.74	0.87	0.78	0.03	0.46	1.00
	0.409	0.588	0.001	0.007	0.463	0.007	0.057	0.000	0.432	0.256	0.000	0.000	0.000	0.000	0.887	0.009	---

Table B

For stem biomass, correlation matrix between all measured cell wall compositional features and with enzymatic saccharification efficiencies of glucose (GlcE), xylose (XylE), arabinose (AraE). Pearson correlation coefficients values are presented, within a colour gradient ranging from red (-1) to green (1). *P*-values, for statistically significant correlations (α=0.05; n=32) are highlighted in red. N is a normalised value representing the total epitope abundance for specific mAbs (see main text for details).

	GlcE (% total Glc)	XylE (% total Xyl)	Glucose (% CWM)	Xylose (% CWM)	%DW lignin	Ferulic acid (ester-linked; % CWM)	p-Coumaric acid (ester-linked; % CWM)	Arabinose/Xylose	Acetate (% CWM)	AraE (% total Ara)	N M87 (OD/g CWM)	N M154 (OD/g CWM)	N M144 (OD/g CWM)	N M137 (OD/g CWM)	N BG1 (OD/g CWM)	N M7 (OD/g CWM)	N M38 (OD/g CWM)
**GlcE (% total Glc)**	1.00																

**XylE (% total Xyl)**	0.87	1.00															
	0.000	---															
**Glucose (% CWM)**	0.10	0.18	1.00														
	0.573	0.324	---														
**Xylose (% CWM)**	-0.07	-0.09	0.77	1.00													
	0.709	0.643	0.000	---													
**%DW lignin**	-0.91	-0.70	0.04	0.21	1.00												
	0.000	0.000	0.818	0.246	---												
**Ferulic acid (ester-linked; % CWM)**	0.09	0.17	0.74	0.43	0.10	1.00											
	0.643	0.355	0.000	0.014	0.581	---											
**p-Coumaric acid (ester-linked; % CWM)**	0.10	0.16	0.66	0.31	0.09	0.89	1.00										
	0.597	0.370	0.000	0.087	0.607	0.000	---										
**Arabinose/Xylose**	0.19	0.04	-0.83	-0.48	-0.28	-0.76	-0.71	1.00									
	0.301	0.817	0.000	0.005	0.124	0.000	0.000	---									
**Acetate (% CWM)**	-0.01	-0.22	-0.67	-0.34	-0.18	-0.73	-0.67	0.67	1.00								
	0.964	0.232	0.000	0.057	0.335	0.000	0.000	0.000	---								
**AraE (% total Ara)**	0.75	0.68	0.30	-0.13	-0.72	0.37	0.40	-0.22	-0.22	1.00							
	0.000	0.000	0.091	0.483	0.000	0.038	0.023	0.230	0.220	---							
**N M87 (OD/g CWM)**	0.20	0.04	-0.38	-0.51	-0.36	-0.36	-0.38	0.16	0.42	0.29	1.00						
	0.278	0.831	0.031	0.003	0.043	0.042	0.030	0.385	0.017	0.111	---						
**N M154 (OD/g CWM)**	0.01	-0.08	-0.52	-0.34	0.02	-0.28	-0.38	0.40	0.36	-0.08	0.60	1.00					
	0.964	0.672	0.002	0.060	0.910	0.121	0.031	0.024	0.045	0.665	0.000	---					
**N M144 (OD/g CWM)**	0.07	-0.04	-0.58	-0.38	-0.05	-0.25	-0.39	0.51	0.36	-0.10	0.46	0.95	1.00				
	0.717	0.839	0.001	0.033	0.784	0.165	0.026	0.003	0.042	0.597	0.008	0.000	---				
**N M137 (OD/g CWM)**	-0.06	-0.09	-0.54	-0.35	0.06	-0.31	-0.46	0.42	0.34	-0.17	0.53	0.97	0.95	1.00			
	0.761	0.616	0.001	0.052	0.728	0.081	0.008	0.017	0.058	0.351	0.002	0.000	0.000	---			
**N BG1 (OD/g CWM)**	0.04	-0.11	0.11	0.04	-0.11	-0.01	-0.04	-0.24	0.16	0.19	0.71	0.40	0.21	0.33	1.00		
	0.835	0.546	0.562	0.833	0.558	0.958	0.844	0.193	0.367	0.302	0.000	0.025	0.245	0.067	---		
**N M7 (OD/g CWM)**	0.26	0.11	-0.09	-0.26	-0.26	0.01	0.13	-0.11	0.04	0.40	0.58	0.35	0.21	0.25	0.44	1.00	
	0.149	0.553	0.642	0.157	0.149	0.944	0.487	0.561	0.811	0.023	0.000	0.048	0.246	0.168	0.012	---	
**N M38 (OD/g CWM)**	0.27	0.06	-0.61	-0.60	-0.41	-0.41	-0.41	0.49	0.52	0.17	0.74	0.61	0.61	0.53	0.32	0.55	1.00
	0.141	0.746	0.000	0.000	0.021	0.019	0.019	0.005	0.002	0.351	0.000	0.000	0.000	0.002	0.076	0.001	---

Table C

For leaf and stem biomass, at each sequential extraction step, Pearson correlation coefficients (*r*) and probability (*P*) values are presented for the correlations between enzymatic saccharification efficiencies of glucose (GlcE) and xylose (XylE), with the N-value for specific glycan epitopes: xyloglucans (CCRC-M87); MLG (BG1); Xylans (CCRC-M137, CCRC-M144, CCRC-M154); Pectins (CCRC-M7, CCRC-M38). N designates the normalised value representing the total epitope abundance (OD/g of CWM).

Xyloglucans and MLG

Tissue			Ammonium oxalate M87	Ammonium oxalate BG1	Sodium Carbonate M87	Sodium Carbonate BG1	1M KOH M87	1M KOH BG1	4M KOH M87	4M KOH BG1	Sodium Chlorite M87	Sodium Chlorite BG1	4M KOH PC M87	4M KOH PC BG1
Leaf	GlcE	*r*	0.63	0.13	0.63	0.50	0.50	0.27	0.76	0.65	-0.12	-0.43	-0.08	-0.17
Leaf	GlcE	*P*	***0.00***	0.47	***0.00***	***0.00***	***0.00***	0.14	***0.00***	***0.00***	0.53	***0.01***	0.66	0.35
Leaf	XylE	*r*	0.27	-0.10	0.73	0.61	0.15	-0.11	0.70	0.71	0.12	-0.50	-0.20	-0.04
Leaf	XylE	*P*	0.13	0.59	***0.00***	***0.00***	0.42	0.54	***0.00***	***0.00***	0.52	***0.00***	0.28	0.82
Stem	GlcE	*r*	0.14	-0.36	-0.01	-0.16	0.30	0.00	0.17	0.14	-0.21	0.00	-0.20	-0.09
Stem	GlcE	*P*	0.44	***0.05***	0.98	0.38	0.09	1.00	0.36	0.46	0.25	0.99	0.28	0.64
Stem	XylE	*r*	0.30	-0.30	-0.21	-0.34	0.30	-0.07	-0.06	-0.10	-0.01	-0.05	-0.07	0.06
Stem	XylE	*P*	0.09	0.09	0.24	0.06	0.09	0.71	0.75	0.57	0.98	0.80	0.69	0.75

Xylans

Tissue			Ammonium oxalate M154	Ammonium oxalate M144	Ammonium oxalate M137	Sodium Carbonate M154	Sodium Carbonate M144	Sodium Carbonate M137	1M KOH M154	1M KOH M144	1M KOH M137
Leaf	GlcE	*r*	0.51	0.04	0.41	0.69	0.66	0.66	-0.52	-0.56	-0.54
Leaf	GlcE	*P*	***0.00***	0.82	***0.02***	***0.00***	***0.00***	***0.00***	***0.00***	***0.00***	***0.00***
Leaf	XylE	*r*	0.23	0.11	0.25	0.82	0.78	0.83	-0.45	-0.45	-0.47
Leaf	XylE	*P*	0.21	0.55	0.17	***0.00***	***0.00***	***0.00***	***0.01***	***0.01***	***0.01***
Stem	GlcE	*r*	0.52	0.41	0.82	-0.23	-0.15	-0.26	0.04	0.05	0.04
Stem	GlcE	*P*	***0.00***	***0.02***	***0.00***	0.20	0.42	0.14	0.82	0.77	0.83
Stem	XylE	*r*	0.41	0.19	0.76	-0.36	-0.30	-0.39	0.08	0.10	0.11
Stem	XylE	*P*	***0.02***	0.30	***0.00***	***0.05***	0.10	***0.03***	0.68	0.60	0.55

Tissue			4M KOH M154	4M KOH M144	4M KOH M137	Sodium Chlorite M154	Sodium Chlorite M144	Sodium Chlorite M137	4M KOH PC M154	4M KOH PC M144	4M KOH PC M137
Leaf	GlcE	*r*	0.77	0.79	0.72	-0.20	-0.25	-0.26	-0.08	-0.08	-0.08
Leaf	GlcE	*P*	***0.00***	***0.00***	***0.00***	0.28	0.16	0.14	0.68	0.67	0.67
Leaf	XylE	*r*	0.77	0.76	0.78	-0.32	-0.33	-0.35	-0.18	-0.17	-0.17
Leaf	XylE	*P*	***0.00***	***0.00***	***0.00***	0.07	0.07	***0.05***	0.33	0.34	0.34
Stem	GlcE	*r*	0.18	0.15	0.13	-0.13	-0.16	-0.21	-0.15	-0.13	-0.17
Stem	GlcE	*P*	0.32	0.41	0.48	0.47	0.37	0.26	0.42	0.48	0.34
Stem	XylE	*r*	-0.03	-0.08	-0.08	-0.28	-0.35	-0.35	-0.01	0.00	-0.04
Stem	XylE	*P*	0.89	0.66	0.67	0.12	0.05	0.05	0.95	0.99	0.83

Pectins

Tissue			Ammonium oxalate M7	Ammonium oxalate M38	Sodium Carbonate M7	Sodium Carbonate M38	1M KOH M7	1M KOH M38	4M KOH M7	4M KOH M38	Sodium Chlorite M7	Sodium Chlorite M38	4M KOH PC M7	4M KOH PC M38
Leaf	GlcE	*r*	0.56	0.20	0.59	0.63	0.44	-0.31	0.88	0.74	0.41	-0.24	0.45	-0.06
Leaf	GlcE	*P*	***0.00***	0.27	***0.00***	***0.00***	***0.01***	0.08	***0.00***	***0.00***	***0.02***	0.19	***0.01***	0.76
Leaf	XylE	*r*	0.18	-0.03	0.52	0.79	0.16	-0.31	0.60	0.58	0.09	-0.20	0.17	-0.14
Leaf	XylE	*P*	0.32	0.89	***0.00***	***0.00***	0.38	0.09	***0.00***	***0.00***	0.62	0.27	0.35	0.44
Stem	GlcE	*r*	0.43	-0.03	0.04	-0.27	0.35	0.28	0.19	0.22	-0.03	-0.03	-0.18	-0.07
Stem	GlcE	*P*	***0.01***	0.86	0.83	0.14	***0.05***	0.13	0.30	0.22	0.87	0.89	0.32	0.72
Stem	XylE	*r*	0.33	-0.19	-0.08	-0.38	0.29	0.17	-0.03	0.03	0.06	-0.10	-0.10	0.01
Stem	XylE	*P*	0.06	0.29	0.68	***0.03***	0.10	0.34	0.87	0.87	0.75	0.57	0.57	0.97

Pearson correlation coefficient: 1 to -1
