# Supplementary material for: Desirable plant cell wall traits for higher-quality miscanthus lignocellulosic biomass
Source: Biotechnol Biofuels. 2019 Apr 15;12:85. doi: 10.1186/s13068-019-1426-7 (PMC6463665; doi:10.1186/s13068-019-1426-7)

Additional file 7

Significantly different cell wall compositional features between high and low yielders of glucose (GlcE, A) or xylose (XylE, B). Distribution of biomass compositional measurements are shown as box and whisker plots, and shaded boxes indicate the features where the high and low yielding classes are significantly different (α=0.05). For leaf or stem cell wall material (CWM), the samples were classified as high or low yielders based on the amount of glucose or xylose released upon enzymatic saccharification. High GlcE designates the top 50% of values, whereas Low GlcE designates the lower 50% of values (consult Table S2 for more detail on GlcE and XylE ranks, and other features). For each outlier, the genotype and harvest time is identified (PB, peak biomass; SS, senesced stage). For each compositional trait, a t-test was performed and probability (P) values are shown. Abbreviations: Glc, glucose; Xyl, xylose; FA, ester-linked ferulic acid; pCA, ester-linked p-coumaric acid; Ara/Xyl, arabinose to xylose ratio; OD, optical density; N, normalised value representing the total epitope abundance for specific mAbs. Further information on all used mAbs can be found in Additional file 1.

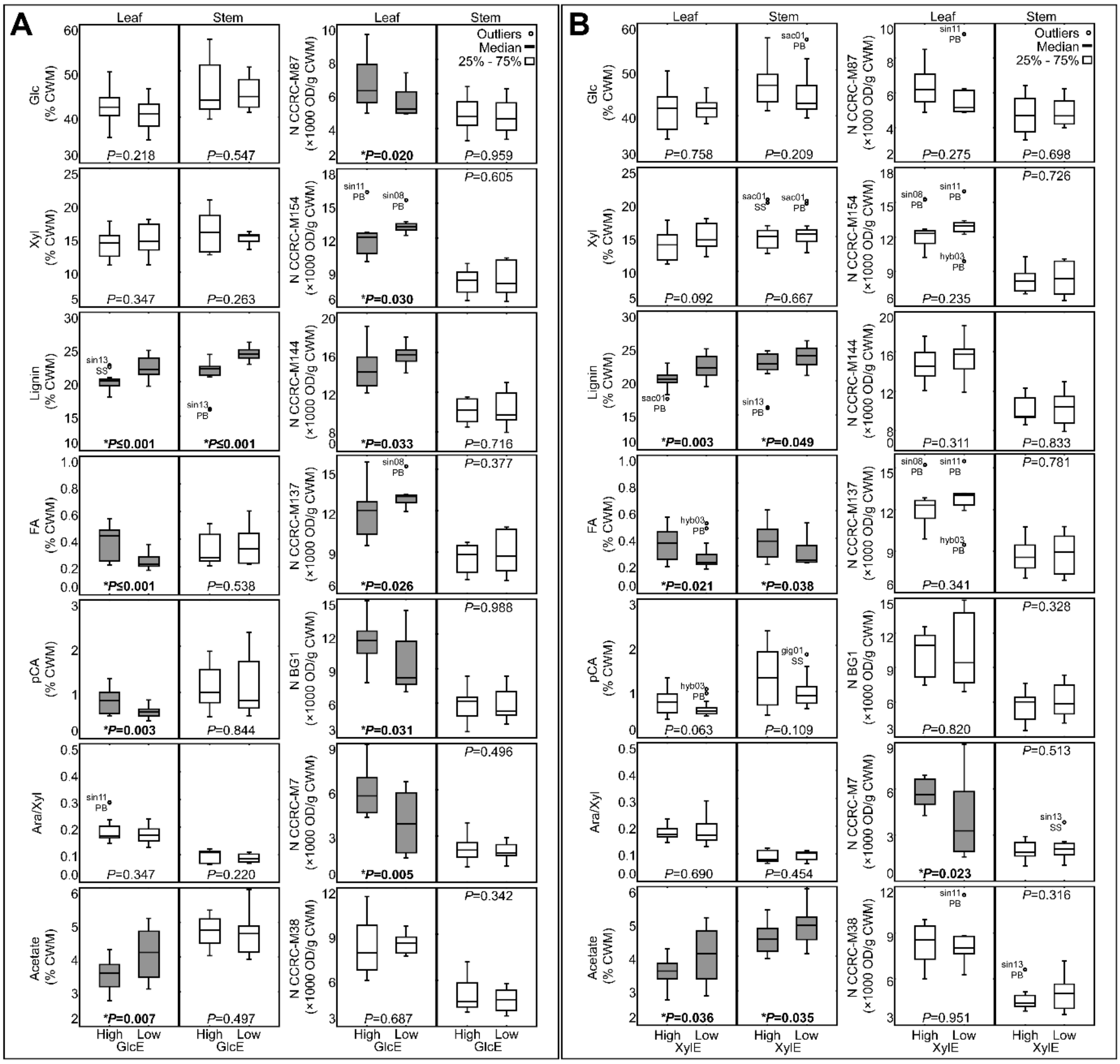

Supplement: Supplementary file 7 — Additional file 7. Box and whisker plots showing the distribution of biomass compositional measurements, and the P-values of t tests used to discriminate between genotypes with high or low saccharification efficiency indices. [file 13068_2019_1426_MOESM7_ESM.pdf]
